# GWAS meta-analysis followed by Mendelian randomization revealed potential control mechanisms for circulating α-Klotho levels

**DOI:** 10.1093/hmg/ddab263

**Published:** 2021-09-20

**Authors:** Ingrid Gergei, Jie Zheng, Till F M Andlauer, Vincent Brandenburg, Nazanin Mirza-Schreiber, Bertram Müller-Myhsok, Bernhard K Krämer, Daniel Richard, Louise Falk, Sofia Movérare-Skrtic, Claes Ohlsson, George Davey Smith, Winfried März, Jakob Voelkl, Jonathan H Tobias

**Affiliations:** Vth Department of Medicine (Nephrology, Hypertensiology, Rheumatology, Endocrinology, Diabetology), University Medical Center, Medical Faculty Mannheim, University of Heidelberg, Mannheim 69117, Germany; Therapeutic Area Cardiovascular Medicine, Boehringer Ingelheim International GmbH, Ingelheim 06877, Germany; MRC Integrative Epidemiology Unit (IEU), Bristol Medical School, University of Bristol, Oakfield House, Oakfield Grove, Bristol BS8 2BN, UK; Population Health Science, Bristol Medical School, University of Bristol, Bristol BS8 2BN, UK; Max Planck Institute of Psychiatry, Munich 80804, Germany; Department of Neurology, Klinikum rechts der Isar, School of Medicine, Technical University of Munich, Munich 80333, Germany; Department of Cardiology and Nephrology, Rhein-Maas Klinikum Würselen, Würselen 52146, Germany; Institute of Neurogenomics, Helmholtz Zentrum München, Neuherberg 85764, Germany; Max Planck Institute of Psychiatry, Munich 80804, Germany; Munich Cluster for Systems Neurology (SyNergy), Munich 2145, Germany; Institute of Translational Medicine, University of Liverpool, Liverpool 11341, UK; Vth Department of Medicine (Nephrology, Hypertensiology, Rheumatology, Endocrinology, Diabetology), University Medical Center, Medical Faculty Mannheim, University of Heidelberg, Mannheim 69117, Germany; European Center for Angioscience ECAS, Medical Faculty Mannheim, University of Heidelberg, Mannheim 69117, Germany; Center for Preventive Medicine and Digital Health Baden-Württemberg (CPDBW), Medical Faculty Mannheim, Heidelberg University, Mannheim 69117, Germany; Department of Human Evolutionary Biology, Harvard University, Cambridge 02138, MA, USA; MRC Integrative Epidemiology Unit (IEU), Bristol Medical School, University of Bristol, Oakfield House, Oakfield Grove, Bristol BS8 2BN, UK; Department of Internal Medicine and Clinical Nutrition, University of Gothenburg, Sahlgrenska Osteoporosis Centre, CBAR, Institute of Medicine, Gothenburg 41296, Sweden; Department of Internal Medicine and Clinical Nutrition, University of Gothenburg, Sahlgrenska Osteoporosis Centre, CBAR, Institute of Medicine, Gothenburg 41296, Sweden; Department of Drug Treatment, Region Västra Götaland, Sahlgrenska University Hospital, Gothenburg 7163, Sweden; MRC Integrative Epidemiology Unit (IEU), Bristol Medical School, University of Bristol, Oakfield House, Oakfield Grove, Bristol BS8 2BN, UK; Population Health Science, Bristol Medical School, University of Bristol, Bristol BS8 2BN, UK; Vth Department of Medicine (Nephrology, Hypertensiology, Rheumatology, Endocrinology, Diabetology), University Medical Center, Medical Faculty Mannheim, University of Heidelberg, Mannheim 69117, Germany; SYNLAB Academy, SYNLAB Holding Deutschland GmbH, Mannheim 24496, Germany; Clinical Institute of Medical and Chemical Laboratory Diagnostics, Medical University of Graz, Graz 8010, Austria; Institute for Physiology and Pathophysiology, Johannes Kepler University Linz, Linz 4040, Austria; Department of Nephrology and Medical Intensive Care, Charité-Universitätsmedizin Berlin, Berlin 10117, Germany; DZHK (German Centre for Cardiovascular Research), Partner Site Berlin, Berlin 10623, Germany; MRC Integrative Epidemiology Unit (IEU), Bristol Medical School, University of Bristol, Oakfield House, Oakfield Grove, Bristol BS8 2BN, UK; Musculoskeletal Research Unit, Translational Health, Learning and Research Building, Level 1 , Southmead Hospital, Bristol BS10 5NB, UK

## Abstract

The protein α-Klotho acts as transmembrane co-receptor for fibroblast growth factor 23 (FGF23) and is a key regulator of phosphate homeostasis. However, α-Klotho also exists in a circulating form, with pleiotropic, but incompletely understood functions and regulation. Therefore, we undertook a genome-wide association study (GWAS) meta-analysis followed by Mendelian randomization (MR) of circulating α-Klotho levels.

Plasma α-Klotho levels were measured by enzyme-linked immunosorbent assay (ELISA) in the Ludwigshafen Risk and Cardiovascular Health and Avon Longitudinal Study of Parents and Children (mothers) cohorts, followed by a GWAS meta-analysis in 4376 individuals across the two cohorts.

Six signals at five loci were associated with circulating α-Klotho levels at genome-wide significance (*P* < 5 × 10^−8^), namely *ABO, KL, FGFR1*, and two post-translational modification genes, *B4GALNT3* and *CHST9*. Together, these loci explained >9% of the variation in circulating α-Klotho levels. MR analyses revealed no causal relationships between α-Klotho and renal function, FGF23-dependent factors such as vitamin D and phosphate levels, or bone mineral density. The screening for genetic correlations with other phenotypes followed by targeted MR suggested causal effects of liability of Crohn’s disease risk [Inverse variance weighted (IVW) beta = 0.059 (95% confidence interval 0.026, 0.093)] and low-density lipoprotein cholesterol levels [−0.198 (−0.332, −0.063)] on α-Klotho.

Our GWAS findings suggest that two enzymes involved in post-translational modification, *B4GALNT3* and *CHST9*, contribute to genetic influences on α-Klotho levels, presumably by affecting protein turnover and stability. Subsequent evidence from MR analyses on α-Klotho levels suggest regulation by mechanisms besides phosphate-homeostasis and raise the possibility of cross-talk with FGF19- and FGF21-dependent pathways, respectively. **Significance statement:** α-Klotho as a transmembrane protein is well investigated along the endocrine FGF23-α-Klotho pathway. However, the role of the circulating form of α-Klotho, which is generated by cleavage of transmembrane α-Klotho, remains incompletely understood. Genetic analyses might help to elucidate novel regulatory and functional mechanisms. The identification of genetic factors related to circulating α-Klotho further enables MR to examine causal relationships with other factors. The findings from the first GWAS meta-analysis of circulating α-Klotho levels identified six genome-wide significant signals across five genes. Given the function of two of the genes identified, *B4GALNT3* and *CHST9,* it is tempting to speculate that post-translational modification significantly contributes to genetic influences on α-Klotho levels, presumably by affecting protein turnover and stability.

## Introduction

α-Klotho is a transmembrane protein that serves with fibroblast growth factor receptors as a co-receptor for fibroblast growth factor 23 (FGF23) (1). The endocrine FGF23-α-Klotho pathway plays a critical role in regulating vitamin D metabolism and phosphate balance (2). Deletion of α-Klotho in mice results in disturbed phosphate homeostasis with an accelerated ageing phenotype, including a shortened lifespan, vascular calcification, infertility and osteoporosis (3). A soluble form of α-Klotho is generated by cleavage of transmembrane α-Klotho, which is readily detected in the circulation, and henceforth referred to as circulating α-Klotho (4). Though the role of circulating α-Klotho remains incompletely understood, it is thought to share functional similarities with the membrane-bound form and to contribute to the actions of its ligand, FGF23 (5). However, circulating α-Klotho also mediates effects independent of FGF23 (6). Previous studies have investigated the clinical utility of serum α-Klotho as a prognostic marker. For example, a recent meta-analysis found that serum levels of circulating α-Klotho are positively related to the estimated glomerular filtration rate (eGFR) in patients with chronic kidney disease (CKD) (7), suggesting a role of α-Klotho as a biomarker for CKD progression. In addition, α-Klotho supplementation is under investigation as a possible drug target for treatment in CKD (5,8).

Genetic studies may prove useful in identifying novel mechanisms which regulate α-Klotho. Several *KL* (*α-klotho*) gene polymorphisms have previously been reported in association with urolithiasis, cardiovascular disease, cancers and longevity (9,10). However, little is known of the genetic pathways which regulate levels of circulating α-Klotho. To our knowledge, a genome-wide association study (GWAS) of α-Klotho has not previously been undertaken. As well as helping to elucidate novel regulatory mechanisms, identification of genetic factors related to circulating α-Klotho enables Mendelian randomization (MR) to examine causal relationships with other factors. This approach can also be used to validate potential drug targets such as α-Klotho, on the basis that the target in question will only modify the outcome (e.g. CKD) in the presence of a causal relationship (11,12).

Therefore, to elucidate novel regulatory mechanisms and functional relationships of circulating α-Klotho, we performed, to our knowledge, the first GWAS meta-analysis of plasma α-Klotho. Subsequently, we used our GWAS output to examine causal relationships between plasma α-Klotho and CKD, other FGF23-dependent pathways [i.e. vitamin D, phosphate and bone mineral density (BMD)], as well as other characteristics with evidence of genetic correlation.

## Results

### GWAS meta-analysis results

**Figure 1:**
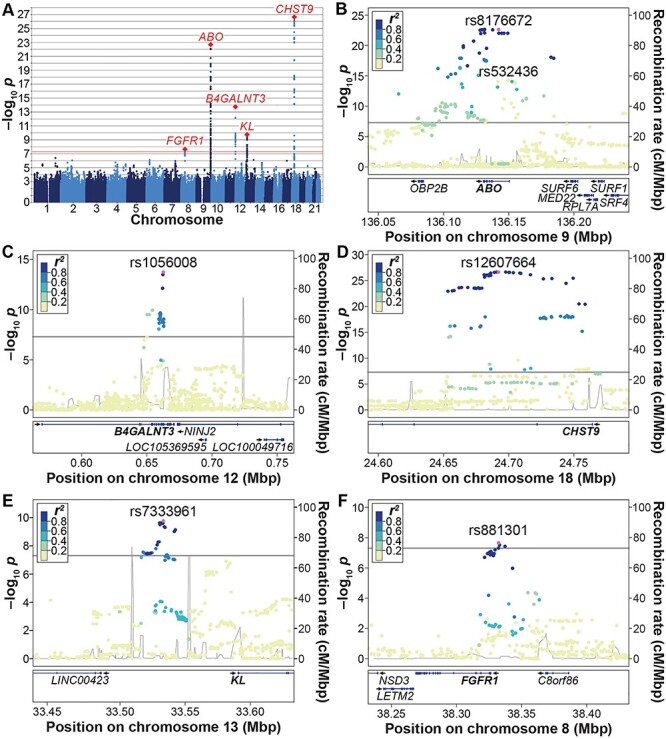
Results from the α-Klotho GWAS meta-analysis. (**A**) Manhattan plot. The *x*-axis indicates the chromosomal position of each SNP, whereas the *y*-axis denotes the evidence of association shown as −log_10_(*P*-value). The red line indicates genome-wide significance of association (*P* = 5 × 10^−8^). (**B**–**F**) Locus-specific Manhattan plots of the genome-wide significant loci at (B) the *ABO* locus, (C) the *B4GALNT3* locus, (D) the *CHST9* locus, (E) the *KL* locus and (F) the *FGFR1* locus. The *x*-axis indicates the physical position of each SNP on the chromosome, the *y*-axis denotes the evidence of association as the −log_10_(*P*-value). The linkage disequilibrium (LD) *r*^2^ between SNPs, based on the 1000 Genomes EUR superpopulation, is shown in colour.

A total of 4376 individuals provided results for the α-Klotho GWAS meta-analysis after combining Ludwigshafen Risk and Cardiovascular Health (LURIC) and Avon Longitudinal Study of Parents and Children (ALSPAC) mothers (Supplementary Material, Table S1). Six independent signals at five loci showed genome-wide significance (Fig. 1, Table 1 and Supplementary Material, Table S3) (median genomic inflation λ = 1.013). The top-associated single-nucleotide polymorphism (SNP), rs12607664 [standard deviations difference relative to mean in plasma α-Klotho per minor allele (MA) T: β = 0.24, standard error (SE) = 0.02, *P* = 2.3 × 10^−27^], mapped to the second intron of the gene *CHST9* on chromosome 18. The second top-associated variant rs8176672 (MA T: β = 0.41, SE = 0.04, *P* = 2.1 × 10^−23^) mapped to the first intron of the *ABO* gene on chromosome 9. A strong association was also observed for another conditionally independent variant at this locus, rs532436, not in linkage disequilibrium (LD) with rs8176672 (*r*^2^ = 0.02 in the 1000 Genome Europeans). A variant at a further locus, rs1056008 (MA C: β = 0.18, SE = 0.02, *P* = 1.8 × 10^−14^), is located in the longest exon of *B4GALNT3* on chromosome 12. We also identified SNPs rs7333961 (MA A: β = −0.33, SE = 0.05, *P* = 1.73 × 10^−10^), upstream of the gene coding for α-Klotho on chromosome 13, and rs881301 (MA C: β = −0.12, SE = 0.02, *P* = 2.2 × 10^−08^), upstream of *FGFR1* on chromosome 8. Genetic associations were broadly similar in both cohorts, with rs8176672 and rs12607664 reaching genome-wide significance in each (Supplementary Material, Fig. S1). That said, *Z*-score test suggested differences between the two cohort in the case of rs12607664, effect size being stronger in ALSPAC. This did not appear to reflect sex differences between the two cohorts, as associations at this locus were identical in LURIC males and females, suggesting other differences are likely responsible such as age.

**Table 1:** Top-associated SNPs of the meta-analysis of both GWAS on plasma α-Klotho levels

SNP	Chr.	EA	OA	EAF	Gene	β	SE	*P*	Pair-wise *Z*	*P*_*Z*
rs12607664	18	T	G	31.61	*CHST9*	0.243	0.022	2.28 × 10^−27^	3.337	0.001
rs8176672	9	T	C	7.18	*ABO*	0.406	0.041	2.11 × 10^−23^	1.785	0.074
rs532436	9	G	A	23.99	*ABO*	0.204	0.026	5.86 × 10^−15^	0.691	0.490
rs1056008	12	C	T	26.82	*B4GALNT3*	0.184	0.024	1.80 × 10^−14^	0.478	0.632
rs7333961	13	A	G	4.62	*KL*	−0.327	0.051	1.73 × 10^−10^	0.816	0.415
rs881301	8	C	T	41.28	*FGFR1*	−0.119	0.021	2.23 × 10^−08^	0.087	0.931

The effect size (β) represents SD difference in α-Klotho relative to the mean (i.e. *Z*-score) per effect allele. Results show univariate/simple linear regression estimates for the six conditionally independent SNPs. The effect size (β) represents SD difference in α-Klotho relative to the mean (i.e. *Z*-scores) per effect allele. Chr., chromosome; EA, effect allele; OA, other allele; EAF, effect allele frequency in the pooled sample in percentage; SE, standard error. Pair-wise *Z* and *P*_*Z* are the pair-wise *Z* estimating the difference between two effect estimates (in LURIC and ALSPAC) and *P*-value of the *Z*-score.

### LD score regression

We used our α-Klotho GWAS data in LD score regression to identify other traits with which α-Klotho might be functionally related, using *P* < 0.05 to indicate findings for potential follow-up, which applied to eight traits (Table 2). Of these, one metabolic trait was related to lipid levels, corresponding to the action of FGF21 (1), which, together with FGF19 and FGF23, comprise the endocrine FGFs (Table 2). In addition, one trait, Crohn’s disease, was related to the action of FGF19 (1).

### MR analyses

Our α-Klotho instruments for MR were based on six SNPs that showed conditional independent effects on α-Klotho using GCTA conditional and joint analysis (COJO) (13), which together accounted for a relatively high proportion of variance (9.1%) (Supplementary Material, Table S4). An *F*-test revealed the instrument to have an acceptable instrumental strength for subsequent MR analyses (*F*-statistic = 74.8). Using these α-Klotho-associated SNPs as instruments of MR, IVW analyses showed no causal effect of α-Klotho on CKD risk (based on an eGFR <60 ml/min/1.73 m^2^) or eGFR derived from either creatinine or cystatin-C (Table 3). Bi-directional analyses using results from eGFR (creatinine) and eGFR (cystatin-C) GWASs as the exposures and our α-Klotho results as the outcome revealed no reverse causality. Null results were also observed in sensitivity analyses (Supplementary Material, Table S5). Similarly, MR analyses examining relationships between α-Klotho and outcomes related to the FGF23 pathway, namely BMD, vitamin D and phosphate levels, did not support causal relationships, including bi-directional and sensitivity analyses (Table 3 and Supplementary Material, Table S6).

Given findings from LD score regression suggesting that α-Klotho might have functional relationships with other endocrine FGFs, we extended our MR analyses to traits related to FGF19 and FGF21. We found no causal effect of α-Klotho on inflammatory bowel disease. However, in bi-directional MR, IVW analyses revealed evidence of a positive relationship for genetic liability to inflammatory bowel disease and, particularly, Crohn’s disease on α-Klotho levels (Table 3), with similar findings in sensitivity analyses (Supplementary Material, Table S7).

IVW and sensitivity analyses revealed no causal relationship between α-Klotho and body mass index (BMI), lipids and traits related to insulin sensitivity (Tables 3, Supplementary Material, Tables S8 and S9). Bi-directional analyses likewise revealed no cause effect of BMI and traits related to insulin sensitivity on α-Klotho. However, IVW suggested a negative causal effect of low-density lipoprotein cholesterol (LDL-C) on α-Klotho, whereas no consistent causal effects were seen for high-density lipoprotein cholesterol (HDL-C) or triglycerides (TGs) (Table 3). The putative causal effect of LDL-C was strengthened when adjusting for HDL-C and TGs in multivariable MR analyses including all three variables (Model 1, Table 4). Additional models examined the contribution of apolipoproteins to these effects, in light of their role in mediating effects of lipoprotein lipids on coronary heart disease (14). In a further multivariable model to study the role of apolipoprotein B (ApoB) (Model 2), only ApoB showed a potentially causal relationship (Table 4). A final multivariable model to study the role of apolipoprotein A-I (ApoA1) (Model 3) found no additional causal effect of ApoA1.

**Table 2:** LD score regression between α-Klotho and human traits (for associations with *P* < 0.05)

Trait 1	Trait 2	*r*_g_	SE	*Z*	*P*
α-Klotho	Excessive frequent and irregular menstruation	0.48	0.21	2.3	0.02
α-Klotho	Fibroblastic disorders	−0.38	0.17	−2.2	0.03
α-Klotho	Triglycerides in small VLDL	−0.54	0.24	−2.2	0.03
α-Klotho	Chronotype	−0.17	0.08	−2.1	0.03
α-Klotho	Crohn’s disease	0.25	0.12	2.1	0.03
α-Klotho	Eczema	−0.48	0.23	−2.1	0.04
α-Klotho	Co-codamol	0.44	0.21	2.1	0.04
α-Klotho	Prostate cancer	0.49	0.25	2.0	0.05

*r*_g_, genetic correlation; SE, standard error of the genetic correlation analysis; *Z*, corresponding *Z*-score; *P*, corresponding *P*-value; VLDL, very low-density lipoprotein.

**Table 3:** Bi-directional MR results between α-Klotho- and FGF-related outcomes

Outcome	MR of α-Klotho versus outcomes			Reverse MR of outcomes versus α-Klotho		
	β	95% CI	*P*	β	95% CI	*P*
Renal outcomes						
CKD	−0.019	−0.077 to 0.039	0.525	−0.170	−0.337 to −0.004	0.055 0.387
eGFR (crea)	0.000	−0.003 to 0.004	0.871	0.405	−0.512 to 1.322	
eGFR (cys)	0.005	−0.291 to 0.300	0.976	0.381	−0.436 to 1.198	0.361
FGF23 pathway outcomes						
eBMD	0.020	−0.017 to 0.056	0.288	0.022	−0.068 to 0.113	0.626
Phosphate	0.009	−0.007 to 0.025	0.288	0.085	−0.073 to 0.243	0.291
Vitamin D	−0.001	−0.018 to 0.015	0.861	0.150	−0.003 to 0.304	0.055
FGF19 pathway outcomes						
IBD	−0.030	−0.109 to 0.049	0.454	0.059	0.026 to 0.093	5.4 × 10^−4^
UC	−0.055	−0.151 to 0.041	0.261	0.039	−0.007 to 0.085	0.099
CD	0.011	−0.102 to 0.124	0.848	0.044	0.003 to 0.086	0.034
FGF21 pathway lipids/obesity						
BMI	−0.010	−0.029 to 0.009	0.304	−0.080	−0.228 to 0.067	0.286
LDL-C	−0.097	−0.237 to 0.044	0.178	−0.198	−0.332 to −0.063	3.9 × 10^−3^
HDL-C	0.018	−0.033 to 0.070	0.490	−0.063	−0.133 to 0.007	0.078
TG	0.006	−0.031 to 0.043	0.748	0.032	−0.087 to 0.152	0.594
Apo A1	−0.006	−0.068 to 0.056	0.843	0.004	−0.030 to 0.037	0.833
Apo B	−0.046	−0.159 to 0.068	0.430	−0.023	−0.052 to 0.007	0.130
FGF21 pathway insulin sensitivity						
Fasting glucose	−0.010	−0.040 to 0.020	0.520	0.186	−0.108 to 0.481	0.215
Fasting insulin	0.009	−0.008 to 0.026	0.296	0.829	−0.702 to 2.360	0.288
HbA1C	−0.019	−0.043 to 0.005	0.120	0.158	−0.266 to 0.583	0.465
HOMA-B	0.015	−0.012 to 0.042	0.279	−0.264	−1.035 to 0.508	0.503

The effect size (β) represents SD change in outcome per SD change in exposure. eGFR (crea), estimated glomerular filtration rate by creatinine plasma concentration; eGFR (cys), estimated glomerular filtration rate by Cystatin C plasma concentration; BMD, bone mineral density; IBD, inflammatory bowel disease; UC, ulcerative colitis; CD, Crohn’s disease; BMI, body mass index; LDL, low-density lipoprotein; HDL, high-density lipoprotein; TG, triglycerides; ApoA1, Apolipoprotein A1; ApoB, Apolipoprotein B; HbA1C, glycated haemoglobin A; HOMA-I, homeostasis model assessment.

**Table 4:** Multivariable MR analyses for lipid traits on α-Klotho

Model	Exposure	Outcome	*N* SNPs	β	SE	*P*
Model 1	LDL-C	α-Klotho	309	−0.173	0.060	3.6 × 10^−3^
	HDL-C	α-Klotho	309	−0.069	0.043	0.106
	TG	α-Klotho	309	0.042	0.065	0.513
Model 2	Low-density lipoprotein	α-Klotho	365	0.070	0.236	0.766
	Triglyceride	α-Klotho	365	−0.227	0.216	0.295
	Apolipoprotein B	α-Klotho	365	0.114	0.056	0.041
Model 3	High-density lipoprotein	α-Klotho	459	−0.070	0.135	0.601
	Apolipoprotein A-I	α-Klotho	459	0.025	0.152	0.870

Model refers to the three multivariable MR models used to examine relationship between lipids and α-Klotho. The effect size (β) represents SD change in outcome per SD change in exposure.

N SNPs, the number of SNPs used in the genetic instrument for the exposure; LDL, low-density lipoprotein; HDL, high-density lipoprotein; TG, triglycerides; β, causal effect size of each lipid subtype on α-Klotho; SE respective standard error; P, respective *p*-value.

### Functional follow-up of GWAS results

According to the expression quantitative trait loci (eQTL) databases GTEx v8 and eQTLGen, rs8176672 and rs532436 were strong *cis*-eQTLs for *ABO* in multiple tissues (Supplementary Material, Table S10). Likewise, rs1056008 and rs881301 were strong eQTLs for *B4GALNT3* and *FGFR1*, respectively, in multiple tissues and both databases. SNP rs12607664 was, according to GTEx, an eQTL for *CHST9* in the cerebellum only. Variant rs7333961 was an eQTL for *KL* in whole blood in the eQTLGen project. Co-localization analyses confirmed a common genetic signal in the case of plasma α-Klotho and *B4GALNT3* eQTL data in whole blood, but the same was not observed for the other loci (Supplementary Material, Table S11). The *B4GALNT3* SNP, rs1056008, had a RegulomeDB score of 1B, indicating a strong likelihood of affecting transcription factor binding and gene expression. No top association signal, or one in high LD, intersected with a DNase-hypersensitive site in the *B4GALNT3* gene in kidney tubule cells.

To explore the contribution of B4GALNT3 to the regulation of circulating α-Klotho levels, α-Klotho levels were measured in *B4galnt3*-deficient mice. However, no clear differences were observed when comparing wild-type, heterozygous and homozygous animals (Supplementary Material, Fig. S2).

## Discussion

Having performed a GWAS for circulating levels of α-Klotho, we identified six GWAS significant signals, mapping to five genes, which together explained over 9% of the variance. This provided genetic predictors of α-Klotho with acceptable strength to interrogate causal relationships. We applied a two-sample MR approach in large GWAS data sets. Despite the potential role of circulating α-Klotho as an early biomarker of CKD (7), MR analyses revealed no causal relationship between circulating α-Klotho and renal function. We also interrogated potential causal relationships between α-Klotho and other outcomes linked to the FGF23 pathway, such as BMD, phosphate and vitamin D, again with little MR evidence. Based on findings from genetic correlation analyses, we also examined causal relationships between α-Klotho and traits related to other endocrine FGFs. Of the six genome-wide significant signals identified in our GWAS meta-analysis, the *cis*-eQTL signal for *B4GALNT3* co-localized with plasma α-Klotho, suggesting that higher levels of *B4GALNT3* expression lead to greater α-Klotho levels. RegulomeDB predicted that our top variant at this locus, rs1056008, alters the binding affinity of activating transcription factors.

As to the mechanisms underlying this genetic association, *B4GALNT3* expresses the enzyme beta-1,4-*N*-acetylgalactosaminyltransferase 3 (EC:2.4.1.244), localizing to the Golgi apparatus. This enzyme transfers *N*-acetylgalactosamine (GalNAc) onto *N*-acetylglucosamine-beta-benzyl to form GalNAcβ1,4-GlcNAc structures on protein epitopes, also known as *N*,*N*′-diacetyllactosediamine (LacdiNAc) (15). We previously found that *B4GALNT3* is expressed at the highest levels in renal tissue (16), an important site of α-Klotho production (8). The presence of the LacdiNAc moiety on circulating proteins such as α-Klotho might influence protein levels in the circulation by altering their turnover and degradation. Such a mechanism may explain why mutations in another glycosylation enzyme, GALNT3, lead to heritable tumoural calcinosis as a consequence of FGF23 deficiency (17). On the other hand, we found that *B4GALNT3* null mice showed no clear alteration in α-Klotho levels in an established immunoprecipitation–immunoblot (IP-IB) assay. One potential explanation for this apparent discrepant finding is that, rather than altering actual levels of α-Klotho, genetic alterations in LacdiNAc content affect the epitope binding with the human ELISA assay used in our study. Furthermore, different functional effects of B4GALNT3-dependent α-Klotho modification in mice and humans cannot be ruled out.

*CHST9* expresses the enzyme carbohydrate sulphotransferase 9, which catalyzes the transfer of a sulphate to terminal LacdiNAc sequences. The two enzymes, B4GALNT3 and CHST9, thus both contribute to generating the terminal SO4-4-GalNAcβ1,4GlcNAcβ structure (18). Several studies have shown that oligosaccharides terminating with this structure are recognized by asialoglycoprotein and mannose receptors and are rapidly degraded (19–22). Such specific receptors have been identified on hepatocytes and endothelial cells (20,21). Interestingly, the clearance rate mediated by the mannose receptor differs by the position of GalNAcβ1,4GlcNAcβ sulphation (23).

The identification of α-Klotho GWAS signals in the *KL* and *FGFR1* genes, coding for the two components of the fibroblast growth factor receptor, was predictable and supports the overall validity of our findings. The *ABO* gene, also found to be associated with α-Klotho in our GWAS, codes for two proteins localizing to the Golgi apparatus, alpha 1–3-*N*-acetylgalactosaminyltransferase (EC:2.4.1.40) and alpha 1–3-galactosyltransferase (EC:2.4.1.37). These enzymes add UDP-*N*-acetyl-galactosamine and UDP-galactose, respectively, to glycoprotein fucosyl-galactosyl residuals (24). Conceivably, α-Klotho may serve as a target for these enzymes, thereby altering its turnover and degradation, as also postulated for B4GALNT3 and CHST9. However, associations with the *ABO* locus, observed in many previous GWASs including a recent GWAS of severe coronavirus disease 2019 with respiratory failure (25), may also have arisen as a result of population stratification despite our best attempts to adjust for this.

Whereas little causal effect was observed for α-Klotho, bi-directional analyses revealed causal effects of genetic liability of Crohn’s disease risk and LDL-C on circulating α-Klotho. Moreover, on multivariable MR of lipid indices, a causal relationship was only retained for ApoB, consistent with the suggestion from another recent multivariable MR that ApoB underlies the relationship between lipid traits and coronary heart disease risk (14). We are not aware of any previous reports linking genetic liability to Crohn’s disease risk or LDL-C to α-Klotho levels. That said, our results are consistent with previous findings that α-Klotho expression is downregulated in hyperlipidaemic mouse models and oxidized-LDL treated tubular cells (26,27). There was reasonably strong statistical evidence for the causal effects of Crohn’s disease which we observed, even when taking into account the multiple traits examined in our MR analyses and the bi-directional causal effects which were evaluated. On the other hand, statistical evidence with respect to causal effects of LDL-C was somewhat weaker having adjusted for multiple comparisons. In the absence of other sources of evidence, further confirmation is required in the case of both of these novel putative causal pathways for α-Klotho levels.

Crohn’s disease and metabolic traits were selected for MR analysis on the basis of their relationship with endocrine FGFs. However, FGF19 and FGF21 pathways (involved in Crohn’s disease and lipid metabolism, respectively) are mediated by β-Klotho, as opposed to α-Klotho (1). Though there is currently no other evidence linking α-Klotho to FGF19 and FGF21 pathways, the present findings certainly raise this as a possibility. Alternatively, relationships between Crohn’s disease susceptibility, LDL-C and circulating α-Klotho which we observed may be independent of endocrine FGFs. It is well recognized that circulating α-Klotho exerts a number of effects independently of FGF23, such as inhibition of insulin, Wingless and Int-1 or transforming growth factor-β signalling (6). In addition, as well as having anti-inflammatory effects by suppressing the transcription factor nuclear factor kappa-light-chain-enhancer of activated B cells (28), α-Klotho renal expression has been found to be downregulated in mouse models of colitis, which was prevented by neutralizing antibodies against Tumor necrosis factor (TNF)-α (29). Accordingly, renal α-Klotho expression is reduced by tumor necrosis factor-like weak inducer of apoptosis and TNF-α (30), and circulating α-Klotho levels are negatively correlated with circulating markers of inflammation (31).

Many of the genetic factors associated with Crohn’s disease risk represent inflammatory mediators, such as IL-23 (32), which could conceivably also affect α-Klotho levels. This raises the possibility of horizontal pleiotropy, whereby genetic factor(s) related to Crohn’s disease affect α-Klotho levels directly, as opposed to via Crohn’s disease risk. That said, sensitivity analyses such as the MR–Egger intercept test did not suggest that pleiotropy contributed to our results, although we recognize that this test is often underpowered.

The main limitation of the present study is the relatively small GWAS sample size. That said, the genetic signals which we identified seemed plausible and provided a relatively strong genetic instrument for subsequent MR analyses. In addition, other characteristics which we examined in relation to α-Klotho were supported by well-powered GWASs that were derived from large data sets. In terms of other limitations, the commercial ELISA employed to measure α-Klotho in the GWAS samples has inferior performance compared with the IP-IB assay, though the latter is unsuitable for use in large cohorts due to its labour-intensive nature (33).

In conclusion, we present findings from the first GWAS of circulating α-Klotho levels, in which we identified six genome-wide significant signals across five genes. Given the function of two of the genes identified, *B4GALNT3* and *CHST9,* it is tempting to speculate that post-translational modification contributes to genetic influences on α-Klotho levels, presumably by affecting protein turnover and stability. In subsequent MR analyses, we found no causal relationship between α-Klotho and CKD or FGF23-dependent pathways. However, there was evidence of a causal effect of Crohn’s disease risk and, to a lesser extent, LDL levels on α-Klotho levels, pointing to novel interactions which require further study.

## Materials and Methods

### LURIC

The LURIC study is a prospective cohort study of individuals with and without cardiovascular disease and was designed to investigate environmental and genetic risk factors for the development of cardiovascular diseases. Between July 1997 and January 2000, 3316 participants of German ancestry were enrolled in the cardiology unit of a tertiary care medical centre in Southwestern Germany. The inclusion criteria were defined as clinical stability except for acute coronary syndromes (ACS), German ancestry and availability of a coronary angiogram (indicated after standard clinical test diagnoses like chest pain and a positive, non-invasive stress test). Exclusion criteria were pre-specified as any acute illness other than ACS, any chronic disease where non-cardiac disease predominated and a history of malignancy within the past 5 years. The detailed study protocol has been published (34). Written informed consent was obtained from each participant prior to inclusion. The study was in accordance with the Declaration of Helsinki and approved by the ethics committee at the Medical Association of Rhineland-Palatinate (Ärztekammer Rheinland-Pfalz). Genotyping was conducted on the Affymetrix 6.0 platform and genotype calling using the algorithm Birdseed v2, both at the LURIC study non-profit LLC, Heidelberg. Quality control was performed in PLINK v1.90b3s (35), as described before (36). Genotype data were imputed to the 1000 Genomes Phase 1 reference panel using SHAPEIT2 and IMPUTE2 (37–39). The resulting data set contained 8 014 018 high-quality variants with an minor allele frequency (MAF) ≥1% and an information metric (INFO) metric ≥0.8 (Supplementary Material S1: Supplementary Methods: LURIC genotype data for more details).

### ALSPAC

ALSPAC is a prospective birth cohort that recruited pregnant women with expected delivery dates between April 1991 and December 1992 from Bristol, UK. The initial number of pregnancies enrolled was 14 541 (for these, at least one questionnaire has been returned or a ‘Children in Focus’ clinic had been attended by July 19, 1999). Of these initial pregnancies, there was a total of 14 676 fetuses, resulting in 14 062 live births and 13 988 children who were alive at 1 year of age. Detailed information on the health and development of children and their parents were collected from regular clinic visits and completion of questionnaires (40,41). Ethical approval was obtained from the ALSPAC Law and Ethics Committee and the Local Ethics Committees. Please note that the study website contains details of all the data that is available through a fully searchable data dictionary (http://www.bristol.ac.uk/alspac/researchers/our-data/). Genotyping of ALSPAC samples was conducted on the Illumina Human660W-Quad platform and genotype calling using Illumina GenomeStudio. Quality control was carried out in PLINK v1.90 (35). Genotype data were imputed to the Haplotype Reference Consortium V1.0 reference panel using SHAPEIT v2 and IMPUTE v2.2.2 (37–39) The resulting data set contained 7 122 422 variants with an MAF ≥1% and INFO ≥0.8.

### Measurement of α-Klotho levels

α-Klotho was measured in plasma samples from both cohorts using the human circulating α-Klotho assay kit (Immuno-Biological Laboratories Co., Ltd, Japan) (42). The lower detection limit was 6.15 pg/ml with a measurement range from 93.75 to 6000 pg/ml. The coefficient of variation was 11.4% at 165.47 pg/ml and 2.9% at 2903.01 pg/ml.

### α-Klotho levels in B4GALNT3 null mice

*B4galnt3* ^−/−^ mice were generated by breeding *B4galnt3*^tm1c(EUCOMM)Wtsi^ male mice (Institut Clinique de la Souris, Illkirch, France), having *LoxP* sites introduced upstream of exon 8 and downstream of exon 9 of the *B4galnt3* gene, with female mice expressing cre recombinase under the control of the phosphoglycerate kinase-1 promoter (PGKcre) (43). The offspring were heterozygous *B4galnt3^+^*/^−^ mice. To generate *B4galnt3*^−/−^ knockout mice*,* heterozygous *B4galnt3*^+/−^, and littermate wild type control mice, female and male *B4galnt3^+/−^* mice were mated. The mice were housed in a standard animal housing facility with a 12 h dark/light period. Food and water were available ad libitum. Before termination at 13 weeks of age, the mice were given an intraperitoneal injection with Ketalar (Pfizer, New York, NY, USA) and Dexdomitor (Orion Pharma, Esbo, Finland) before they were bled and euthanized with cervical dislocation. The animal experiments were approved by the Ethics Committee at University of Gothenburg, and the care of the animals was according to their guidelines. Circulating α-Klotho in mouse serum of *B4galnt3* wild-type, heterozygous and knockout mice was measured by an established IP-IB assay at the UT Southwestern (TX, USA) (44). Differences between groups were analysed by the Steel–Dwass test.

### GWAS meta-analysis

α-Klotho levels were transformed using rank-based inverse normal transformation before analysis. GWAS was conducted using linear regression on imputed probabilities in PLINK for LURIC and SNPTEST for ALSPAC data. Sex, age and the first eight ancestry principle components (PCs) were used as covariates in LURIC, where sex, age and the first 10 PCs were used as covariates in ALSPAC. The final sample sizes were *n* = 2234 for LURIC and *n* = 2142 for ALSPAC (Supplementary Material, Table S1). Results from both GWAS were combined using fixed-effects meta-analysis in METAL (45), the pooled data set containing 6 439 450 common variants and *N* = 4376 individuals. A threshold of *P* < 5 × 10^−8^ was used to denote genome-wide significance. A *Z*-score test, comparing mean estimates of associations between the two cohorts, was used to evaluate heterogeneity.

### Conditional analysis

To detect multiple independent association signals at each of the genome-wide significant α-Klotho loci, we carried out an approximate conditional and joint genome-wide association analysis using the software package GCTA-COJO (13). SNPs in LD (LD *r*^2^ > 0.9) were ignored, and those situated >10 Mb away were assumed to be in complete linkage equilibrium. A reference sample of 8890 unrelated individuals of ALSPAC mothers was used to model patterns of LD between variants. The reference genotyping data set consisted of the same 6.44 million variants assessed in our GWAS meta-analysis. Conditionally independent variants that reached GWAS significance were annotated to the physically closest gene with the hg19 gene range list available in dbSNP (https://www.ncbi.nlm.nih.gov/SNP/).

### LD score regression

Two analyses were conducted using LD score regression (46,47). First, we quantified the overall SNP-based heritability using LD score regression utilizing a subset of 1.2 million HapMap SNPs (with INFO >0.9 and MAF ≥1%). Second, we estimated the genetic correlation between plasma α-Klotho levels and 832 human traits implemented in the LD Hub database (48). This method uses the cross-products of summary test statistics from two GWASs and regresses them against a measure of how much variation each SNP tags (i.e. its LD score). Variants with high LD scores are more likely to contain more true signals and thus provide a greater chance of overlap with genuine signals between GWASs.

### MR

We examined the evidence for a causal relationship between plasma α-Klotho levels and several renal phenotypes using two-sample MR (49). Primary analyses were performed using the inverse-variance weighted method, followed by sensitivity analyses [MR–Egger, weighted median, simple mode and weighted mode (50–52)]. Bi-directional analyses were also performed to examine reverse causality, where α-Klotho was considered as the outcome. In addition, MR analyses examined causal relationships between α-Klotho and other FGF23-dependent pathways and phenotypes, namely BMD, vitamin D and phosphate.

LD score regression suggested possible relationships with IBD and metabolic outcomes, with which the other endocrine FGFs, FGF19 and FGF21, have previously been implicated (1). Therefore, MR analyses also examined relationships between α-Klotho and inflammatory bowel disease, and between α-Klotho and metabolic outcomes. In total, 24 outcomes were selected for the MR analysis (Supplementary Material, Table S2 for GWAS sources). After adjusting the influence of correlations among the 24 outcomes using PhenoSpD (53), 14 independent variables/tests remained. Therefore, in this further set of MR analyses, the threshold corrected for multiple testing using Bonferroni’s method was defined as α = 0.05/14 = 0.004. This figure was then divided by two to account for the bi-directional analyses performed for each outcome, giving a final figure of 0.002.

In addition, given lipid traits are correlated with each other, multivariable MR (54) was conducted for five lipid traits on α-Klotho to control for such a correlation. Three multivariable MR models were used:

1. LDL-C, HDL-C and TG versus α-Klotho.
2. LDL-C, TG and APOB versus α-Klotho.
3. HDL-C and APOA1 versus α-Klotho.

### Genetic functional analyses

*Cis*-acting effects of the genome-wide significant GWAS signals were examined by lookups in the GTEx database v8 (55) and the eQTLGen database (56). Regulatory elements in non-coding regions of the human genome were identified using RegulomeDB v1.1 (57). To further estimate whether the top association signals of α-Klotho and gene eQTLs of the *cis*-genes share the same causal variants within a 1 Mb window around the top signals of the α-Klotho GWAS, we used a Bayesian model (*coloc*) to estimate such posterior probabilities (PP) (58). The default priors for co-localization analyses were used (the prior probability a SNP is associated with α-Klotho was 1 × 10^−4^; the prior probability a SNP is associated with gene expression of the *cis*-gene was 1 × 10^−4^; and the prior probability a SNP is associated with both α-Klotho and gene expression was 1 × 10^−5^). A lack of evidence (i.e. a PP <80%) in the co-localization analysis goes against the hypothesis that the *cis*-gene mediates the effect of the genetic signal in question on α-Klotho levels. The gene expression data were extracted from the eQTLGen database. We treated co-localized findings (PP ≥80%) as ‘colocalised’ and other results that did not pass co-localization as ‘not colocalised’.

DNase-I hypersensitivity site data was obtained from the ENCODE database (59) or two replicates of human kidney tubule primary cell cultures (accessions: ENF428WYR and ENCFF711TUV). Called peaks were lifted-over to hg19 using the liftOver utility (60) with ‘minMatch = 0.1’, all other settings left to default. Peaks were considered replicable if present in both samples—the middles of overlapping peaks were padded to define 150 bp regions. Genome-wide significant SNPs (defined as reported *P*-value <5e-8) were intersected with regions using bedtools intersect (61). In addition, assay for transposase-accessible chromatin using sequencing open-chromatin regions from mouse embryonic (E15.5) distal femur growth plate (GEO accession GSM2687479) (62) were obtained and lifted-over to hg19 using the liftOver utility as above. Whether genome-wide significant SNPs overlapped with these regions was then examined using bedtools.

## Authors’ Contribution

I.G., J.Z., T.F.M.A., N.M.S., D.R., L.F., S.M.S., C.O., W.M., J.V. and J.H.T. contributed to the concept, interpretation, critical writing and/or analysis of the data. All authors contributed to revision of the intellectual content and final approval of the version to be published.

## Supplementary Material

Supplementary_Material_final-v2_ddab263
