# Supplementary material for: GWAS meta-analysis followed by Mendelian randomization revealed potential control mechanisms for circulating α-Klotho levels
Source: Hum Mol Genet. 2021 Sep 20;31(5):792–802. doi: 10.1093/hmg/ddab263 (PMC8895756; doi:10.1093/hmg/ddab263)
Supplement: Supplementary_Material_final-v2_ddab263 [file supplementary_material_final-v2_ddab263.docx]

**GWAS META-ANALYSIS FOLLOWED BY MENDELIAN RANDOMISATION SUGGESTS CAUSAL EFFECTS OF CROHN’S DISEASE RISK AND LDL CHOLESTEROL ON CIRCULATING α-KLOTHO**

**Supplementary Material**

*Table of Contents*

[Supplementary Methods: LURIC genotype data 2](#_Toc78640929)

[Genetic quality control (QC) and imputation 2](#_Toc78640930)

[Sequence of genotype QC 2](#_Toc78640931)

[Imputation of genotype data 2](#_Toc78640932)

[Calculation of MDS ancestry components 2](#_Toc78640933)

[Supplementary Tables 3](#_Toc78640934)

[Supplementary Table S1: Cohort characteristics 3](#_Toc78640935)

[Supplementary Table S2: GWAS sources for two-sample MR studies 4](#_Toc78640936)

[Supplementary Table S3: Top SNPs of the meta-analysis of both GWAS on plasma α-Klotho levels 5](#_Toc78640937)

[Supplementary Table S4: α-Klotho instruments used in the MR analysis 6](#_Toc78640938)

[Supplementary Table S5: Bidirectional MR analyses for α-Klotho on renal function 7](#_Toc78640939)

[Supplementary Table S6: Bidirectional MR analyses for α-Klotho on outcomes related to the FGF23 pathway: BMD, phosphate and vitamin D 9](#_Toc78640940)

[Supplementary Table S7: Bidirectional MR analyses for α-Klotho on inflammatory bowel disease 11](#_Toc78640941)

[Supplementary Table S8: Bidirectional MR analyses for α-Klotho on BMI and lipids 13](#_Toc78640942)

[Supplementary Table S9: Bidirectional MR analyses for α-Klotho on diabetes-related traits 16](#_Toc78640943)

[Supplementary Table S10: *Cis*-eQTL lookup for the top α-Klotho association signals across multiple tissues from the GTEx consortium 19](#_Toc78640944)

[Supplementary Table S11: Colocalisation analyses between α-Klotho and eQTLs of the genes closest to the top GWAS SNPs 22](#_Toc78640945)

[Supplementary Figures 23](#_Toc78640946)

[Supplementary Figure S1: Forest Plots 23](#_Toc78640947)

[Supplementary Figure S2: Open-chromatin results 24](#_Toc78640948)

[Supplementary Figure S3: α-Klotho levels in *B4GALNT3* null mice 24](#_Toc78640949)

# Supplementary Methods: LURIC genotype data

## Genetic quality control (QC) and imputation

QC of genotype data was conducted in PLINK v1.90b3s and *R* v3.3.3.

### Sequence of genotype QC

**Before QC:** 3,061 individuals and 687,262 variants

- 1. Removal of SNPs with call rates <98% or a minor allele frequency (MAF) <1%
  2. Removal of individuals with genotyping rates <98%
  3. Removal of sex mismatches
  4. Removal of genetic duplicates
  5. Removal of cryptic relatives with PI-HAT≥1/8
  6. Removal of genetic outliers with a distance from the mean of >4.3 SD in the first eight MDS (ancestry) components
  7. Removal of individuals with a deviation of the autosomal or X-chromosomal heterozygosity from the mean >4 SD
  8. Removal of non-autosomal variants
  9. Removal of SNPs with call rates <98%, a MAF <1%, or Hardy-Weinberg Equilibrium (HWE) test *p*-values <1×10^-6^
  10. Removal of A/T and G/C SNPs
  11. Update of variant IDs and positions to the IDs and positions in the 1000 Genomes Phase 1 reference panel
  12. Alignment of alleles to the reference panel
  13. Removal of duplicated variants and variants not present in the reference panel

**After QC:** 2,839 individuals and 556,894 variants

### Imputation of genotype data

Genotypes were aligned to the 1000 Genomes Phase 1 reference panel using SHAPEIT v2 and PLINK. Pre-phasing (haplotype estimation) was conducted for each chromosome separately using SHAPEIT. Imputation was performed using IMPUTE2 in 5 Mbp chunks with 500 kbp buffers, filtering out variants that are monomorphic in the EUR samples. Chunks with <51 genotyped variants or concordance rates <92 % were fused with neighboring chunks and re-imputed. Variants with a MAF <1% or an INFO metric <0.8 were removed after imputation.

### Calculation of MDS ancestry components

For the calculation of multidimensional scaling (MDS) ancestry components (population stratification), pre-imputation genotype data was used, after the QC steps explained above had been applied. Additional variant filtering steps were: removal of variants with a MAF <0.05 or HWE *p*‑value <10^‑3^; removal of variants mapping to the extended MHC region (chromosome 6, 25-35 Mbp) or to a typical inversion site on chromosome 8 (7‑13 Mbp); LD pruning (command *--indep-pairwise 200 100 0.2*). Next, the pairwise identity-by-state (IBS) matrix of all individuals was calculated using the command *‑‑genome* on the filtered genotype data. The MDS analysis was performed on the IBS matrix using the eigendecomposition-based algorithm in PLINK. MDS components calculated for each cohort separately were used as covariates (the ancestry components) in analyses using genetic data.

# Supplementary Tables

## Supplementary Table S1: Cohort characteristics

| Cohort | N α-Klotho | N GWAS | Mean age (SD) | Female (%) |
| --- | --- | --- | --- | --- |
| LURIC | 2615 | 2234 | 62.3 (10.5) | 31.6 |
| ALSPAC | 2330 | 2142 | 47.9 (4.4) | 100 |

N α-Klotho = total sample size with α-Klotho measurements

N GWAS = total sample size with α-Klotho measurements and linked genotype data

## Supplementary Table S2: GWAS sources for two-sample MR studies

| Category | Phenotype name | Abbreviation | PMID / BioRxiv | Consortium |
| --- | --- | --- | --- | --- |
| Kidney function | Chronic kidney disease | CKD | 31152163 | CKDGen |
|  | Estimated Glomerular Filtration Rate (creatinine-based) | eGFRcrea | 31152163 | CKDGen |
|  | Estimated Glomerular Filtration Rate (cystatin C-based) | eGFRcys | 27920155 | CKDGen |
| Body weight | Body mass index | BMI | 25673413 | GIANT |
|  | Whole body fat mass | Body fat | https://gwas.mrcieu.ac.uk | UKBB |
| Protein | Sclerostin | SOST | 31170332 | Zheng *et al.* |
| Lipids | HDL cholesterol | HDL-C | 24097068 | GLGC |
|  | LDL cholesterol | LDL-C | 24097068 | GLGC |
|  | Triglycerides | TG | 24097068 | GLGC |
|  | Apolipoprotein A-I | APOA1 | 32203549 | UKBB |
|  | Apolipoprotein B | APOB | 32203549 | UKBB |
| Inflammatory bowel disease | Crohn's disease | CD | 26192919 | IBDGenetics |
|  | Inflammatory bowel disease | IBD | 26192919 | IBDGenetics |
|  | Ulcerative colitis | UC | 26192919 | IBDGenetics |
| Glycemic trait | 2hr glucose | 2hG | 20081857 | MAGIC |
|  | HbA1C \|\| id:758 | HbA1C | 20858683 | MAGIC |
|  | Fasting proinsulin \|\| id:769 | FP | 21873549 | MAGIC |
|  | HOMA-B \|\| id:770 | HOMA-B | 20081858 | MAGIC |
|  | HOMA-IR \|\| id:771 | HOMA-IR | 20081858 | MAGIC |
|  | Fasting glucose \|\| id:773 | FG | 22885924 | MAGIC |
|  | Fasting insulin \|\| id:775 | FI | 20081858 | MAGIC |
| Vitamin | Vitamin-D | VD | 32242144 | Revez *et al.* |
| Multiple sclerosis | Multiple sclerosis | MS | 31604244 | IMSGC |
| Phosphate | Phosphate | PHO | https://gwas.mrcieu.ac.uk | UKBB |
| Bone | Estimate bone mineral density | eBMD | 30598549 | UKBB |

## Supplementary Table S3: Top SNPs of the meta-analysis of both GWAS on plasma α-Klotho levels (conditional SNP effects)

| **SNP** | **Chr.** | **EA** | **OA** | **EAF** | **Gene** | **β** | **SE** | **P** |
| --- | --- | --- | --- | --- | --- | --- | --- | --- |
| rs12607664 | 18 | T | G | 31.61 | *CHST9* | 0.24 | 0.02 | 1.15×10-26 |
| rs8176672 | 9 | T | C | 7.18 | *ABO* | 0.37 | 0.04 | 2.80×10-19 |
| rs532436 | 9 | G | A | 23.99 | *ABO* | 0.17 | 0.03 | 5.23×10-11 |
| rs1056008 | 12 | C | T | 26.82 | *B4GALNT3* | 0.18 | 0.02 | 2.39×10-14 |
| rs7333961 | 13 | A | G | 4.62 | *KL* | -0.33 | 0.05 | 2.03×10-10 |
| rs881301 | 8 | C | T | 41.28 | *FGFR1* | -0.12 | 0.02 | 2.46×10-08 |

SNP effects of the six conditionally independent signals: β, β_se and β_P represent the conditional SNP effects of the six signals. The effect size (β) represents SD difference in α-Klotho relative to the mean per effect allele. EA, effect allele; OA, other allele; EAF, effect allele frequency in the pooled sample in %; se, standard error.

## Supplementary Table S4: α-Klotho instruments used in the MR analysis

| SNP | Chr. | Position (hg19) | Reference allele | Allele frequency | β | SE | P-value | N | Variance explained |
| --- | --- | --- | --- | --- | --- | --- | --- | --- | --- |
| rs881301 | 8 | 38332318 | T | 0.587 | 0.118 | 0.021 | 2.23E-08 | 4559.9 | 0.682% |
| rs8176672 | 9 | 136142185 | T | 0.071 | 0.406 | 0.041 | 2.11E-23 | 4431.3 | 2.197% |
| rs532436 | 9 | 136149830 | A | 0.214 | -0.204 | 0.026 | 5.86E-15 | 4263.9 | 1.405% |
| rs1056008 | 12 | 662838 | T | 0.731 | -0.183 | 0.024 | 1.80E-14 | 4402.6 | 1.321% |
| rs7333961 | 13 | 33533269 | A | 0.046 | -0.327 | 0.051 | 1.73E-10 | 4289.3 | 0.943% |
| rs12607664 | 18 | 24693221 | T | 0.316 | 0.242 | 0.022 | 2.28E-27 | 4494.0 | 2.542% |

SNP, Chr., Position, Reference allele, and Allele frequency are the rsID, chromosome and position (hg19), reference allele, and allele frequency of the genetic instruments. β, SE, and P-value are the effect size, standard error and *p*-value of the SNP association on α-Klotho. N is the sample size of each SNP. Variance explained is the phenotypic variance explained by each SNP.

## Supplementary Table S5: Bidirectional MR analyses for α-Klotho on renal function

| exposure | outcome | method | nSNP | β | SE | P | Q | DF_Q_ | P_Q_ | Int_Egger_ | SE_Egger_ | P_Egger_ |
| --- | --- | --- | --- | --- | --- | --- | --- | --- | --- | --- | --- | --- |
| Klotho | CKD | Inverse variance weighted | 5 | -0.019 | 0.029 | 0.525 | 6.533 | 4 | 0.163 |  |  |  |
| Klotho | CKD | MR Egger | 5 | 0.040 | 0.080 | 0.649 | 5.386 | 3 | 0.146 | -0.015 | 0.018 | 0.483 |
| Klotho | CKD | Weighted median | 5 | -0.024 | 0.032 | 0.448 |  |  |  |  |  |  |
| Klotho | CKD | Simple mode | 5 | -0.049 | 0.050 | 0.383 |  |  |  |  |  |  |
| Klotho | CKD | Weighted mode | 5 | -0.040 | 0.045 | 0.421 |  |  |  |  |  |  |
| Klotho | eGFRcrea | Inverse variance weighted | 5 | 0.000 | 0.002 | 0.871 | 17.121 | 4 | 0.002 |  |  |  |
| Klotho | eGFRcrea | MR Egger | 5 | -0.003 | 0.005 | 0.540 | 14.038 | 3 | 0.003 | 0.001 | 0.001 | 0.476 |
| Klotho | eGFRcrea | Weighted median | 5 | 0.001 | 0.001 | 0.445 |  |  |  |  |  |  |
| Klotho | eGFRcrea | Simple mode | 5 | 0.002 | 0.002 | 0.421 |  |  |  |  |  |  |
| Klotho | eGFRcrea | Weighted mode | 5 | 0.001 | 0.002 | 0.535 |  |  |  |  |  |  |
| Klotho | eGFRcys | Inverse variance weighted | 6 | 0.005 | 0.151 | 0.976 | 19.322 | 5 | 0.002 |  |  |  |
| Klotho | eGFRcys | MR Egger | 6 | -0.867 | 0.220 | 0.017 | 1.466 | 4 | 0.833 | 0.204 | 0.048 | 0.013 |
| Klotho | eGFRcys | Weighted median | 6 | -0.031 | 0.106 | 0.770 |  |  |  |  |  |  |
| Klotho | eGFRcys | Simple mode | 6 | 0.002 | 0.157 | 0.992 |  |  |  |  |  |  |
| Klotho | eGFRcys | Weighted mode | 6 | -0.011 | 0.139 | 0.938 |  |  |  |  |  |  |
| eGFRcys | Klotho | Inverse variance weighted | 4 | 0.381 | 0.417 | 0.361 | 4.475 | 3 | 0.215 |  |  |  |
| eGFRcys | Klotho | MR Egger | 4 | 0.475 | 0.767 | 0.599 | 4.416 | 2 | 0.110 | -0.004 | 0.028 | 0.886 |
| eGFRcys | Klotho | Weighted median | 4 | 0.417 | 0.354 | 0.238 |  |  |  |  |  |  |
| eGFRcys | Klotho | Simple mode | 4 | 1.418 | 0.939 | 0.228 |  |  |  |  |  |  |
| eGFRcys | Klotho | Weighted mode | 4 | 0.393 | 0.372 | 0.368 |  |  |  |  |  |  |
| eGFRcrea | Klotho | Inverse variance weighted | 41 | 0.405 | 0.468 | 0.387 | 41.125 | 40 | 0.421 |  |  |  |
| eGFRcrea | Klotho | MR Egger | 41 | 2.520 | 1.589 | 0.121 | 39.180 | 39 | 0.462 | -0.018 | 0.013 | 0.172 |
| eGFRcrea | Klotho | Weighted median | 41 | 0.750 | 0.706 | 0.288 |  |  |  |  |  |  |
| eGFRcrea | Klotho | Simple mode | 41 | 1.376 | 1.273 | 0.286 |  |  |  |  |  |  |
| eGFRcrea | Klotho | Weighted mode | 41 | 1.514 | 1.103 | 0.177 |  |  |  |  |  |  |

nSNP is the number of SNPs used used in the MR analyses. β, SE, and P are the centre estimate, standard error, and *p*-value of the MR estimates. Q, DF_Q_, and P_Q_ are the test statistcs, degrees of freedom, and *p*-value of Cohorane’s Q test. Int_Egger_, SE_Egger_, and P_Egger_ are the intercept, standard error, and *p*-value of the MR-Egger intercept test.

## Supplementary Table S6: Bidirectional MR analyses for α-Klotho on outcomes related to the FGF23 pathway: BMD, phosphate and vitamin D

| exposure | outcome | method | nSNP | β | SE | P | Q | DF_Q_ | P_Q_ | Int_Egger_ | SE_Egger_ | P_Egger_ |
| --- | --- | --- | --- | --- | --- | --- | --- | --- | --- | --- | --- | --- |
| Vitamin-D | klotho | Inverse variance weighted | 97 | 0.150 | 0.078 | 0.055 | 111.35 | 96 | 0.135 |  |  |  |
| Vitamin-D | klotho | MR Egger | 97 | 0.077 | 0.101 | 0.445 | 109.85 | 95 | 0.141 | 0.004 | 0.004 | 0.257 |
| Vitamin-D | klotho | Weighted median | 97 | 0.128 | 0.102 | 0.207 |  |  |  |  |  |  |
| Vitamin-D | klotho | Simple mode | 97 | 0.067 | 0.255 | 0.792 |  |  |  |  |  |  |
| Vitamin-D | klotho | Weighted mode | 97 | 0.131 | 0.083 | 0.118 |  |  |  |  |  |  |
| Phosphate | klotho | Inverse variance weighted | 115 | 0.085 | 0.081 | 0.291 | 110.73 | 114 | 0.569 |  |  |  |
| Phosphate | klotho | MR Egger | 115 | 0.035 | 0.142 | 0.804 | 110.54 | 113 | 0.548 | 0.002 | 0.004 | 0.670 |
| eBMD | klotho | MR Egger | 303 | 0.061 | 0.093 | 0.514 | 289.16 | 301 | 0.678 | -0.001 | 0.003 | 0.636 |
| eBMD | klotho | Weighted median | 303 | 0.076 | 0.078 | 0.330 | 289.39 | 302 | 0.689 |  |  |  |
| eBMD | klotho | Inverse variance weighted | 303 | 0.022 | 0.046 | 0.626 |  |  |  |  |  |  |
| eBMD | klotho | Simple mode | 303 | 0.112 | 0.188 | 0.554 |  |  |  |  |  |  |
| eBMD | klotho | Weighted mode | 303 | 0.128 | 0.116 | 0.272 |  |  |  |  |  |  |
| Klotho | Vitamin-D | Inverse variance weighted | 4 | -0.001 | 0.008 | 0.861 | 6.54 | 3 | 0.088 |  |  |  |
| Klotho | Vitamin-D | MR Egger | 4 | -0.017 | 0.021 | 0.503 | 4.912 | 2 | 0.086 | 0.004 | 0.005 | 0.501 |
| Klotho | Vitamin-D | Weighted median | 4 | -0.003 | 0.007 | 0.644 |  |  |  |  |  |  |
| Klotho | Vitamin-D | Simple mode | 4 | -0.009 | 0.011 | 0.467 |  |  |  |  |  |  |
| Klotho | Vitamin-D | Weighted mode | 4 | -0.006 | 0.009 | 0.565 |  |  |  |  |  |  |
| Klotho | Phosphate | Inverse variance weighted | 6 | 0.009 | 0.008 | 0.288 | 9.897 | 5 | 0.078 |  |  |  |
| Klotho | Phosphate | MR Egger | 6 | 0.051 | 0.017 | 0.039 | 2.769 | 4 | 0.597 | -0.010 | 0.004 | 0.056 |
| Klotho | eBMD | MR Egger | 6 | 0.018 | 0.059 | 0.779 | 45.211 | 4 | 3.59E-09 | 0.000 | 0.013 | 0.975 |
| Klotho | eBMD | Weighted median | 6 | 0.009 | 0.009 | 0.312 | 45.224 | 5 | 1.31E-08 |  |  |  |
| Klotho | eBMD | Inverse variance weighted | 6 | 0.020 | 0.018 | 0.288 |  |  |  |  |  |  |
| Klotho | eBMD | Simple mode | 6 | 0.013 | 0.011 | 0.260 |  |  |  |  |  |  |
| Klotho | eBMD | Weighted mode | 6 | 0.007 | 0.010 | 0.484 |  |  |  |  |  |  |

nSNP is the number of SNPs used used in the MR analyses. β, SE, and P are the centre estimate, standard error, and *p*-value of the MR estimates. Q, DF_Q_, and P_Q_ are the test statistcs, degrees of freedom, and *p*-value of Cohorane’s Q test. Int_Egger_, SE_Egger_, and P_Egger_ are the intercept, standard error, and *p*-value of the MR-Egger intercept test.

## Supplementary Table S7: Bidirectional MR analyses for α-Klotho on inflammatory bowel disease

| exposure | outcome | method | nSNP | β | SE | P | Q | DF_Q_ | P_Q_ | Int_Egger_ | SE_Egger_ | P_Egger_ |
| --- | --- | --- | --- | --- | --- | --- | --- | --- | --- | --- | --- | --- |
| Klotho | Crohn's disease | IVW | 6 | 0.011 | 0.057 | 0.848 | 6.02 | 5.00 | 0.30 |  |  |  |
| Klotho | Crohn's disease | MR Egger | 6 | 0.157 | 0.161 | 0.386 | 4.88 | 4.00 | 0.30 | -0.04 | 0.04 | 0.39 |
| Klotho | Crohn's disease | Weighted median | 6 | 0.023 | 0.070 | 0.748 |  |  |  |  |  |  |
| Klotho | Crohn's disease | Simple mode | 6 | -0.096 | 0.129 | 0.492 |  |  |  |  |  |  |
| Klotho | Crohn's disease | Weighted mode | 6 | 0.072 | 0.097 | 0.494 |  |  |  |  |  |  |
| Klotho | IBD | IVW | 6 | -0.030 | 0.040 | 0.454 | 5.43 | 5.00 | 0.37 |  |  |  |
| Klotho | IBD | MR Egger | 6 | 0.124 | 0.109 | 0.317 | 3.13 | 4.00 | 0.54 | -0.04 | 0.02 | 0.20 |
| Klotho | IBD | Weighted median | 6 | -0.034 | 0.048 | 0.485 |  |  |  |  |  |  |
| Klotho | IBD | Simple mode | 6 | -0.127 | 0.093 | 0.233 |  |  |  |  |  |  |
| Klotho | IBD | Weighted mode | 6 | -0.125 | 0.066 | 0.116 |  |  |  |  |  |  |
| Klotho | Ulcerative colitis | IVW | 6 | -0.055 | 0.049 | 0.261 | 2.33 | 5.00 | 0.80 |  |  |  |
| Klotho | Ulcerative colitis | MR Egger | 6 | 0.053 | 0.138 | 0.718 | 1.63 | 4.00 | 0.80 | -0.03 | 0.03 | 0.45 |
| Klotho | Ulcerative colitis | Weighted median | 6 | -0.043 | 0.061 | 0.482 |  |  |  |  |  |  |
| Klotho | Ulcerative colitis | Simple mode | 6 | -0.040 | 0.083 | 0.650 |  |  |  |  |  |  |
| Klotho | Ulcerative colitis | Weighted mode | 6 | -0.030 | 0.076 | 0.712 |  |  |  |  |  |  |
| Crohn's disease | klotho | IVW | 46 | 0.059 | 0.017 | 5.36E-04 | 32.41 | 45.00 | 0.92 |  |  |  |
| Crohn's disease | klotho | MR Egger | 46 | 0.052 | 0.039 | 1.92E-01 | 32.36 | 44.00 | 0.90 | 0.00 | 0.01 | 0.83 |
| Crohn's disease | klotho | Weighted median | 46 | 0.053 | 0.026 | 4.32E-02 |  |  |  |  |  |  |
| Crohn's disease | klotho | Simple mode | 46 | 0.086 | 0.051 | 9.67E-02 |  |  |  |  |  |  |
| Crohn's disease | klotho | Weighted mode | 46 | 0.052 | 0.035 | 1.44E-01 |  |  |  |  |  |  |
| IBD | klotho | IVW | 54 | 0.044 | 0.021 | 3.47E-02 | 37.89 | 53.00 | 0.94 |  |  |  |
| IBD | klotho | MR Egger | 54 | 0.005 | 0.057 | 9.37E-01 | 37.33 | 52.00 | 0.94 | 0.01 | 0.01 | 0.46 |
| IBD | klotho | Weighted median | 54 | 0.059 | 0.033 | 7.54E-02 |  |  |  |  |  |  |
| IBD | klotho | Simple mode | 54 | 0.130 | 0.060 | 3.66E-02 |  |  |  |  |  |  |
| IBD | klotho | Weighted mode | 54 | 0.096 | 0.050 | 5.82E-02 |  |  |  |  |  |  |
| Ulcerative colitis | klotho | IVW | 32 | 0.039 | 0.024 | 9.92E-02 | 23.49 | 31.00 | 0.83 |  |  |  |
| Ulcerative colitis | klotho | MR Egger | 32 | -0.002 | 0.080 | 9.77E-01 | 23.20 | 30.00 | 0.81 | 0.01 | 0.01 | 0.59 |
| Ulcerative colitis | klotho | Weighted median | 32 | 0.044 | 0.032 | 1.68E-01 |  |  |  |  |  |  |
| Ulcerative colitis | klotho | Simple mode | 32 | 0.031 | 0.063 | 6.31E-01 |  |  |  |  |  |  |
| Ulcerative colitis | klotho | Weighted mode | 32 | 0.037 | 0.054 | 5.04E-01 |  |  |  |  |  |  |

nSNP is the number of SNPs used used in the MR analyses. β, SE, and P are the centre estimate, standard error, and *p*-value of the MR estimates. Q, DF_Q_, and P_Q_ are the test statistcs, degrees of freedom, and *p*-value of Cohorane’s Q test. Int_Egger_, SE_Egger_, and P_Egger_ are the intercept, standard error, and *p*-value of the MR-Egger intercept test.

## Supplementary Table S8: Bidirectional MR analyses for α-Klotho on BMI and lipids

| exposure | outcome | method | nSNP | β | SE | P | Q | DF_Q_ | P_Q_ | Int_Egger_ | SE_Egger_ | P_Egger_ |
| --- | --- | --- | --- | --- | --- | --- | --- | --- | --- | --- | --- | --- |
| Klotho | Body mass index | IVW | 5 | -0.010 | 0.010 | 0.304 | 2.74 | 4.00 | 0.60 |  |  |  |
| Klotho | Body mass index | MR Egger | 5 | 0.018 | 0.026 | 0.545 | 1.44 | 3.00 | 0.70 | -0.01 | 0.01 | 0.34 |
| Klotho | Body mass index | Weighted median | 5 | -0.007 | 0.011 | 0.538 |  |  |  |  |  |  |
| Klotho | Body mass index | Simple mode | 5 | -0.007 | 0.016 | 0.668 |  |  |  |  |  |  |
| Klotho | Body mass index | Weighted mode | 5 | -0.007 | 0.014 | 0.626 |  |  |  |  |  |  |
| Klotho | HDL-C | IVW | 5 | 0.018 | 0.026 | 0.490 | 19.05 | 4.00 | 0.00 |  |  |  |
| Klotho | HDL-C | MR Egger | 5 | 0.043 | 0.079 | 0.620 | 18.32 | 3.00 | 0.00 | -0.01 | 0.02 | 0.75 |
| Klotho | HDL-C | Weighted median | 5 | 0.038 | 0.015 | 0.011 |  |  |  |  |  |  |
| Klotho | HDL-C | Simple mode | 5 | 0.044 | 0.017 | 0.059 |  |  |  |  |  |  |
| Klotho | HDL-C | Weighted mode | 5 | 0.041 | 0.016 | 0.056 |  |  |  |  |  |  |
| Klotho | LDL-C | IVW | 5 | -0.097 | 0.072 | 0.178 | 121.50 | 4.00 | 0.00 |  |  |  |
| Klotho | LDL-C | MR Egger | 5 | -0.088 | 0.220 | 0.715 | 121.43 | 3.00 | 0.00 | 0.00 | 0.05 | 0.97 |
| Klotho | LDL-C | Weighted median | 5 | 0.009 | 0.022 | 0.690 |  |  |  |  |  |  |
| Klotho | LDL-C | Simple mode | 5 | 0.007 | 0.026 | 0.805 |  |  |  |  |  |  |
| Klotho | LDL-C | Weighted mode | 5 | 0.012 | 0.021 | 0.596 |  |  |  |  |  |  |
| Klotho | Triglycerides | IVW | 5 | 0.006 | 0.019 | 0.748 | 10.05 | 4.00 | 0.04 |  |  |  |
| Klotho | Triglycerides | MR Egger | 5 | 0.038 | 0.053 | 0.526 | 8.81 | 3.00 | 0.03 | -0.01 | 0.01 | 0.56 |
| Klotho | Triglycerides | Weighted median | 5 | 0.010 | 0.017 | 0.539 |  |  |  |  |  |  |
| Klotho | Triglycerides | Simple mode | 5 | 0.015 | 0.029 | 0.636 |  |  |  |  |  |  |
| Klotho | Triglycerides | Weighted mode | 5 | 0.005 | 0.024 | 0.832 |  |  |  |  |  |  |
| Klotho | APOA1 | MR Egger | 6 | 0.001 | 0.094 | 0.988 | 10.18 | 4.00 | 0.04 | 0.00 | 0.02 | 0.93 |
| Klotho | APOA1 | Weighted median | 6 | 0.000 | 0.029 | 0.989 | 10.21 | 5.00 | 0.07 |  |  |  |
| Klotho | APOA1 | IVW | 6 | -0.006 | 0.032 | 0.843 |  |  |  |  |  |  |
| Klotho | APOA1 | Simple mode | 6 | 0.045 | 0.050 | 0.404 |  |  |  |  |  |  |
| Klotho | APOA1 | Weighted mode | 6 | 0.005 | 0.038 | 0.893 |  |  |  |  |  |  |
| Klotho | APOB | MR Egger | 6 | -0.024 | 0.172 | 0.895 | 36.15 | 4.00 | 0.00 | -0.01 | 0.04 | 0.90 |
| Klotho | APOB | Weighted median | 6 | -0.021 | 0.032 | 0.509 | 36.32 | 5.00 | 0.00 |  |  |  |
| Klotho | APOB | IVW | 6 | -0.046 | 0.058 | 0.430 |  |  |  |  |  |  |
| Klotho | APOB | Simple mode | 6 | 0.018 | 0.043 | 0.694 |  |  |  |  |  |  |
| Klotho | APOB | Weighted mode | 6 | -0.037 | 0.038 | 0.377 |  |  |  |  |  |  |
| body mass index | klotho | MR Egger | 470 | 0.338 | 0.198 | 0.089 | 564.30 | 468.00 | 0.00 | -0.01 | 0.00 | 0.02 |
| body mass index | klotho | Weighted median | 470 | 0.065 | 0.126 | 0.605 | 570.57 | 469.00 | 0.00 |  |  |  |
| body mass index | klotho | IVW | 470 | -0.080 | 0.075 | 0.286 |  |  |  |  |  |  |
| body mass index | klotho | Simple mode | 470 | 0.055 | 0.395 | 0.889 |  |  |  |  |  |  |
| body mass index | klotho | Weighted mode | 470 | 0.086 | 0.220 | 0.695 |  |  |  |  |  |  |
| HDL-C | klotho | IVW | 152 | -0.063 | 0.036 | 0.078 | 176.40 | 151.00 | 0.08 |  |  |  |
| HDL-C | klotho | MR Egger | 152 | -0.068 | 0.049 | 0.170 | 176.38 | 150.00 | 0.07 | 0.00 | 0.00 | 0.89 |
| HDL-C | klotho | Weighted median | 152 | -0.097 | 0.050 | 0.051 |  |  |  |  |  |  |
| HDL-C | klotho | Simple mode | 152 | -0.030 | 0.106 | 0.778 |  |  |  |  |  |  |
| HDL-C | klotho | Weighted mode | 152 | -0.081 | 0.042 | 0.055 |  |  |  |  |  |  |
| APOA1 | klotho | IVW | 319 | 0.004 | 0.017 | 0.833 | 428.97 | 318.00 | 0.00 |  |  |  |
| APOA1 | klotho | MR Egger | 319 | 0.020 | 0.028 | 0.478 | 428.24 | 317.00 | 0.00 | 0.00 | 0.00 | 0.46 |
| APOA1 | klotho | Weighted median | 319 | 0.037 | 0.025 | 0.142 |  |  |  |  |  |  |
| APOA1 | klotho | Simple mode | 319 | 0.026 | 0.060 | 0.667 |  |  |  |  |  |  |
| APOA1 | klotho | Weighted mode | 319 | 0.034 | 0.028 | 0.228 |  |  |  |  |  |  |
| APOB | klotho | IVW | 179 | -0.023 | 0.015 | 0.130 | 173.52 | 178.00 | 0.58 |  |  |  |
| APOB | klotho | MR Egger | 179 | -0.025 | 0.023 | 0.269 | 173.50 | 177.00 | 0.56 | 0.00 | 0.00 | 0.89 |
| APOB | klotho | Weighted median | 179 | -0.013 | 0.023 | 0.584 |  |  |  |  |  |  |
| APOB | klotho | Simple mode | 179 | -0.097 | 0.051 | 0.057 |  |  |  |  |  |  |
| APOB | klotho | Weighted mode | 179 | -0.022 | 0.023 | 0.349 |  |  |  |  |  |  |
| LDL-C | klotho | IVW | 113 | -0.198 | 0.069 | 0.004 | 168.99 | 112.00 | 0.00 |  |  |  |
| LDL-C | klotho | MR Egger | 113 | -0.176 | 0.115 | 0.128 | 168.91 | 111.00 | 0.00 | 0.00 | 0.00 | 0.81 |
| LDL-C | klotho | Weighted median | 113 | -0.056 | 0.092 | 0.545 |  |  |  |  |  |  |
| LDL-C | klotho | Simple mode | 113 | -0.144 | 0.159 | 0.366 |  |  |  |  |  |  |
| LDL-C | klotho | Weighted mode | 113 | -0.076 | 0.080 | 0.346 |  |  |  |  |  |  |
| Triglyceride | klotho | IVW | 126 | 0.032 | 0.061 | 0.594 | 183.64 | 125.00 | 0.00 |  |  |  |
| Triglyceride | klotho | MR Egger | 126 | -0.181 | 0.095 | 0.059 | 172.15 | 124.00 | 0.00 | 0.01 | 0.00 | 0.00 |
| Triglyceride | klotho | Weighted median | 126 | -0.046 | 0.078 | 0.555 |  |  |  |  |  |  |
| Triglyceride | klotho | Simple mode | 126 | -0.087 | 0.128 | 0.496 |  |  |  |  |  |  |
| Triglyceride | klotho | Weighted mode | 126 | -0.124 | 0.070 | 0.079 |  |  |  |  |  |  |

nSNP is the number of SNPs used used in the MR analyses. β, SE, and P are the centre estimate, standard error, and *p*-value of the MR estimates. Q, DF_Q_, and P_Q_ are the test statistcs, degrees of freedom, and *p*-value of Cohorane’s Q test. Int_Egger_, SE_Egger_, and P_Egger_ are the intercept, standard error, and *p*-value of the MR-Egger intercept test.

## Supplementary Table S9: Bidirectional MR analyses for α-Klotho on diabetes-related traits

| exposure | outcome | method | nSNP | β | SE | P | Q | DF_Q_ | P_Q_ | Int_Egger_ | SE_Egger_ | P_Egger_ |
| --- | --- | --- | --- | --- | --- | --- | --- | --- | --- | --- | --- | --- |
| Klotho | 2hr glucose | IVW | 5 | -0.023 | 0.045 | 0.616 | 1.04 | 4.00 | 0.90 |  |  |  |
| Klotho | 2hr glucose | MR Egger | 5 | -0.056 | 0.120 | 0.675 | 0.95 | 3.00 | 0.81 | 0.01 | 0.03 | 0.79 |
| Klotho | 2hr glucose | Weighted median | 5 | -0.042 | 0.054 | 0.436 |  |  |  |  |  |  |
| Klotho | 2hr glucose | Simple mode | 5 | -0.047 | 0.067 | 0.518 |  |  |  |  |  |  |
| Klotho | 2hr glucose | Weighted mode | 5 | -0.047 | 0.068 | 0.533 |  |  |  |  |  |  |
| Klotho | HbA1C | IVW | 5 | -0.019 | 0.012 | 0.120 | 6.69 | 4.00 | 0.15 |  |  |  |
| Klotho | HbA1C | MR Egger | 5 | -0.052 | 0.036 | 0.245 | 5.10 | 3.00 | 0.16 | 0.01 | 0.01 | 0.40 |
| Klotho | HbA1C | Weighted median | 5 | -0.014 | 0.013 | 0.280 |  |  |  |  |  |  |
| Klotho | HbA1C | Simple mode | 5 | -0.023 | 0.019 | 0.289 |  |  |  |  |  |  |
| Klotho | HbA1C | Weighted mode | 5 | -0.016 | 0.015 | 0.333 |  |  |  |  |  |  |
| Klotho | FPI | IVW | 5 | 0.026 | 0.018 | 0.146 | 3.00 | 4.00 | 0.56 |  |  |  |
| Klotho | FPI | MR Egger | 5 | -0.017 | 0.048 | 0.739 | 2.05 | 3.00 | 0.56 | 0.01 | 0.01 | 0.40 |
| Klotho | FPI | Weighted median | 5 | 0.024 | 0.021 | 0.265 |  |  |  |  |  |  |
| Klotho | FPI | Simple mode | 5 | 0.025 | 0.030 | 0.464 |  |  |  |  |  |  |
| Klotho | FPI | Weighted mode | 5 | 0.028 | 0.027 | 0.367 |  |  |  |  |  |  |
| Klotho | HOMA-B | IVW | 5 | 0.015 | 0.014 | 0.279 | 11.77 | 4.00 | 0.02 |  |  |  |
| Klotho | HOMA-B | MR Egger | 5 | 0.073 | 0.023 | 0.049 | 3.36 | 3.00 | 0.34 | -0.01 | 0.01 | 0.07 |
| Klotho | HOMA-B | Weighted median | 5 | 0.024 | 0.010 | 0.016 |  |  |  |  |  |  |
| Klotho | HOMA-B | Simple mode | 5 | 0.026 | 0.011 | 0.067 |  |  |  |  |  |  |
| Klotho | HOMA-B | Weighted mode | 5 | 0.026 | 0.010 | 0.069 |  |  |  |  |  |  |
| Klotho | HOMA-IR | IVW | 5 | 0.005 | 0.015 | 0.721 | 9.08 | 4.00 | 0.06 |  |  |  |
| Klotho | HOMA-IR | MR Egger | 5 | 0.057 | 0.033 | 0.182 | 4.64 | 3.00 | 0.20 | -0.01 | 0.01 | 0.19 |
| Klotho | HOMA-IR | Weighted median | 5 | 0.009 | 0.013 | 0.470 |  |  |  |  |  |  |
| Klotho | HOMA-IR | Simple mode | 5 | 0.008 | 0.017 | 0.668 |  |  |  |  |  |  |
| Klotho | HOMA-IR | Weighted mode | 5 | 0.014 | 0.016 | 0.433 |  |  |  |  |  |  |
| Klotho | Fasting glucose | IVW | 5 | -0.010 | 0.015 | 0.520 | 12.35 | 4.00 | 0.01 |  |  |  |
| Klotho | Fasting glucose | MR Egger | 5 | -0.045 | 0.049 | 0.426 | 10.36 | 3.00 | 0.02 | 0.01 | 0.01 | 0.50 |
| Klotho | Fasting glucose | Weighted median | 5 | -0.012 | 0.013 | 0.357 |  |  |  |  |  |  |
| Klotho | Fasting glucose | Simple mode | 5 | -0.036 | 0.030 | 0.293 |  |  |  |  |  |  |
| Klotho | Fasting glucose | Weighted mode | 5 | 0.016 | 0.017 | 0.388 |  |  |  |  |  |  |
| Klotho | Fasting insulin | IVW | 5 | 0.009 | 0.009 | 0.296 | 2.40 | 4.00 | 0.66 |  |  |  |
| Klotho | Fasting insulin | MR Egger | 5 | 0.033 | 0.025 | 0.282 | 1.38 | 3.00 | 0.71 | -0.01 | 0.01 | 0.39 |
| Klotho | Fasting insulin | Weighted median | 5 | 0.008 | 0.011 | 0.432 |  |  |  |  |  |  |
| Klotho | Fasting insulin | Simple mode | 5 | -0.001 | 0.015 | 0.956 |  |  |  |  |  |  |
| Klotho | Fasting insulin | Weighted mode | 5 | 0.008 | 0.013 | 0.572 |  |  |  |  |  |  |
| 2hr glucose | klotho | Wald ratio | 1 | -0.058 | 0.184 | 0.750 |  |  |  |  |  |  |
| HbA1C | klotho | IVW | 11 | 0.158 | 0.217 | 0.465 | 5.96 | 10.00 | 0.82 |  |  |  |
| HbA1C | klotho | MR Egger | 11 | 0.352 | 0.515 | 0.511 | 5.78 | 9.00 | 0.76 | -0.01 | 0.02 | 0.69 |
| HbA1C | klotho | Weighted median | 11 | 0.052 | 0.280 | 0.853 |  |  |  |  |  |  |
| HbA1C | klotho | Simple mode | 11 | -0.178 | 0.470 | 0.713 |  |  |  |  |  |  |
| HbA1C | klotho | Weighted mode | 11 | -0.149 | 0.332 | 0.664 |  |  |  |  |  |  |
| FPI | klotho | IVW | 8 | -0.123 | 0.107 | 0.248 | 1.64 | 7.00 | 0.98 |  |  |  |
| FPI | klotho | MR Egger | 8 | -0.184 | 0.252 | 0.494 | 1.57 | 6.00 | 0.95 | 0.01 | 0.02 | 0.80 |
| FPI | klotho | Weighted median | 8 | -0.128 | 0.131 | 0.329 |  |  |  |  |  |  |
| FPI | klotho | Simple mode | 8 | -0.060 | 0.187 | 0.756 |  |  |  |  |  |  |
| FPI | klotho | Weighted mode | 8 | -0.099 | 0.150 | 0.532 |  |  |  |  |  |  |
| HOMA-B | klotho | IVW | 3 | -0.264 | 0.394 | 0.503 | 0.46 | 2.00 | 0.79 |  |  |  |
| HOMA-B | klotho | MR Egger | 3 | -0.442 | 2.323 | 0.880 | 0.46 | 1.00 | 0.50 | 0.01 | 0.08 | 0.95 |
| HOMA-B | klotho | Weighted median | 3 | -0.136 | 0.450 | 0.763 |  |  |  |  |  |  |
| HOMA-B | klotho | Simple mode | 3 | -0.085 | 0.538 | 0.889 |  |  |  |  |  |  |
| HOMA-B | klotho | Weighted mode | 3 | -0.046 | 0.527 | 0.939 |  |  |  |  |  |  |
| Fasting glucose | klotho | IVW | 22 | 0.186 | 0.150 | 0.215 | 13.72 | 21.00 | 0.88 |  |  |  |
| Fasting glucose | klotho | MR Egger | 22 | 0.102 | 0.313 | 0.749 | 13.62 | 20.00 | 0.85 | 0.00 | 0.01 | 0.76 |
| Fasting glucose | klotho | Weighted median | 22 | 0.059 | 0.201 | 0.769 |  |  |  |  |  |  |
| Fasting glucose | klotho | Simple mode | 22 | 0.009 | 0.369 | 0.982 |  |  |  |  |  |  |
| Fasting glucose | klotho | Weighted mode | 22 | 0.077 | 0.223 | 0.733 |  |  |  |  |  |  |
| Fasting insulin | klotho | IVW | 3 | 0.829 | 0.781 | 0.288 | 2.69 | 2.00 | 0.26 |  |  |  |
| Fasting insulin | klotho | MR Egger | 3 | 2.749 | 6.069 | 0.729 | 2.43 | 1.00 | 0.12 | -0.04 | 0.13 | 0.80 |
| Fasting insulin | klotho | Weighted median | 3 | 1.473 | 0.860 | 0.087 |  |  |  |  |  |  |
| Fasting insulin | klotho | Simple mode | 3 | 1.662 | 1.189 | 0.297 |  |  |  |  |  |  |
| Fasting insulin | klotho | Weighted mode | 3 | 1.675 | 1.225 | 0.305 |  |  |  |  |  |  |

nSNP is the number of SNPs used used in the MR analyses. β, SE, and P are the centre estimate, standard error, and *p*-value of the MR estimates. Q, DF_Q_, and P_Q_ are the test statistcs, degrees of freedom, and *p*-value of Cohorane’s Q test. Int_Egger_, SE_Egger_, and P_Egger_ are the intercept, standard error, and *p*-value of the MR-Egger intercept test.

## Supplementary Table S10: *Cis*-eQTL lookup for the top α-Klotho association signals across multiple tissues from the GTEx consortium

| Locus | EA/OE | SNP | P-value | β | Tissue |
| --- | --- | --- | --- | --- | --- |
| ABO | C/T | rs891364023 | 4.8e-74 | 1.4 | Skin - Sun Exposed (Lower leg) |
| ABO | C/T | rs891364023 | 1.4e-67 | 1.5 | Skin - Not Sun Exposed (Suprapubic) |
| ABO | C/T | rs891364023 | 8.6e-48 | 1.2 | Whole Blood |
| ABO | C/T | rs891364023 | 3.4e-46 | 1.0 | Thyroid |
| ABO | C/T | rs891364023 | 7.6e-38 | 1.0 | Nerve - Tibial |
| ABO | C/T | rs891364023 | 1.1e-37 | 0.70 | Lung |
| ABO | C/T | rs891364023 | 5.0e-35 | 0.90 | Esophagus - Mucosa |
| ABO | C/T | rs891364023 | 1.6e-33 | 0.92 | Muscle - Skeletal |
| ABO | C/T | rs891364023 | 5.2e-21 | 0.75 | Adipose - Visceral (Omentum) |
| ABO | C/T | rs891364023 | 5.7e-20 | 0.73 | Testis |
| ABO | C/T | rs891364023 | 2.7e-17 | 0.65 | Adipose - Subcutaneous |
| ABO | C/T | rs891364023 | 6.9e-17 | 0.64 | Stomach |
| ABO | C/T | rs891364023 | 2.1e-15 | 0.67 | Esophagus - Muscularis |
| ABO | C/T | rs891364023 | 4.8e-15 | 0.72 | Esophagus - Gastroesophageal Junction |
| OBP2B | C/T | rs891364023 | 1.3e-13 | -0.45 | Skin - Sun Exposed (Lower leg) |
| ABO | C/T | rs891364023 | 2.3e-12 | 1.0 | Brain - Frontal Cortex (BA9) |
| ABO | C/T | rs891364023 | 8.3e-12 | 0.35 | Artery - Tibial |
| ABO | C/T | rs891364023 | 1.5e-11 | 0.89 | Brain - Cortex |
| ABO | C/T | rs891364023 | 3.1e-11 | 0.61 | Heart - Atrial Appendage |
| ABO | C/T | rs891364023 | 3.6e-11 | 0.50 | Heart - Left Ventricle |
| ABO | C/T | rs891364023 | 3.8e-11 | 1.0 | Brain - Anterior cingulate cortex (BA24) |
| ABO | C/T | rs891364023 | 1.9e-10 | 0.57 | Pituitary |
| ABO | C/T | rs891364023 | 3.5e-10 | 0.29 | Colon – Transverse |
| OBP2B | C/T | rs891364023 | 1.0e-9 | -0.60 | Heart - Left Ventricle |
| ABO | C/T | rs891364023 | 3.0e-9 | 0.49 | Colon - Sigmoid |
| ABO | C/T | rs891364023 | 5.1e-9 | 0.70 | Ovary |
| ABO | C/T | rs891364023 | 7.7e-9 | 0.70 | Prostate |
| SURF6 | C/T | rs891364023 | 1.4e-8 | -0.50 | Esophagus - Gastroesophageal Junction |
| ABO | C/T | rs891364023 | 3.0e-8 | 0.77 | Pancreas |
| SURF1 | C/T | rs891364023 | 5.3e-8 | 0.34 | Skin - Sun Exposed (Lower leg) |
| ABO | C/T | rs891364023 | 4.8e-7 | 0.71 | Adrenal Gland |
| ABO | C/T | rs891364023 | 8.3e-7 | 0.40 | Artery – Coronary |
| SURF1 | C/T | rs891364023 | 8.5e-7 | 0.37 | Heart - Atrial Appendage |
| SURF6 | C/T | rs891364023 | 1.0e-6 | -0.25 | Whole Blood |
| SURF6 | C/T | rs891364023 | 1.9e-6 | -0.29 | Artery - Tibial |
| SURF6 | C/T | rs891364023 | 3.0e-6 | -0.23 | Adipose - Visceral (Omentum) |
| ABO | C/T | rs891364023 | 7.0e-6 | 0.48 | Brain - Nucleus accumbens (basal ganglia) |
| SURF6 | C/T | rs891364023 | 1.5 | -0.30 | Esophagus - Muscularis |
| DBH-AS1 | C/T | rs891364023 | 3.4e-5 | 0.29 | Nerve - Tibial |
| ABO | C/T | rs891364023 | 3.8e-5 | 0.45 | Breast - Mammary Tissue |
| MED22 | C/T | rs891364023 | 4.4e-5 | -0.13 | Whole Blood |
| OBP2B | C/T | rs891364023 | 7.9e-5 | -0.21 | Skin - Not Sun Exposed (Suprapubic) |
| SURF1 | C/T | rs891364023 | 8.5e-5 | 0.30 | Esophagus - Mucosa |
| GBGT1 | C/T | rs891364023 | 1.0e-4 | -0.14 | Cells - Cultured fibroblasts |
| ABO | A/G | rs947073006 | 3.9e-33 | 0.75 | Whole Blood |
| ABO | A/G | rs947073006 | 1.8e-25 | -0.56 | Adipose - Visceral (Omentum) |
| ABO | A/G | rs947073006 | 3.9e-24 | -0.52 | Adipose - Subcutaneous |
| ABO | A/G | rs947073006 | 2.3e-18 | -0.47 | Muscle - Skeletal |
| ABO | A/G | rs947073006 | 1.0e-15 | -0.28 | Artery - Tibial |
| ABO | A/G | rs947073006 | 5.6e-14 | -0.38 | Heart - Left Ventricle |
| ABO | A/G | rs947073006 | 8.3e-13 | -0.41 | Esophagus - Muscularis |
| SURF1 | A/G | rs947073006 | 2.0e-12 | 0.38 | Heart - Atrial Appendage |
| ABO | A/G | rs947073006 | 5.9e-12 | -0.37 | Esophagus - Mucosa |
| ABO | A/G | rs947073006 | 9.4e-11 | -0.58 | Adrenal Gland |
| SURF1 | A/G | rs947073006 | 1.7e-10 | 0.35 | Esophagus - Gastroesophageal Junction |
| SURF1 | A/G | rs947073006 | 2.4e-10 | 0.27 | Adipose - Subcutaneous |
| ABO | A/G | rs947073006 | 4.3e-10 | -0.40 | Pituitary |
| ABO | A/G | rs947073006 | 5.1e-10 | -0.45 | Breast - Mammary Tissue |
| SURF1 | A/G | rs947073006 | 5.3e-10 | 0.35 | Colon - Transverse |
| SURF1 | A/G | rs947073006 | 1.2e-9 | 0.24 | Adipose - Visceral (Omentum) |
| ABO | A/G | rs947073006 | 2.6e-9 | -0.68 | Liver |
| SURF1 | A/G | rs947073006 | 2.6e-9 | 0.32 | Heart - Left Ventricle |
| ABO | A/G | rs947073006 | 5.7e-9 | -0.29 | Colon - Sigmoid |
| ABO | A/G | rs947073006 | 8.1e-9 | -0.38 | Heart - Atrial Appendage |
| SURF1 | A/G | rs947073006 | 9.7e-9 | 0.24 | Muscle - Skeletal |
| SURF1 | A/G | rs947073006 | 1.2e-8 | 0.30 | Esophagus – Muscularis |
| ABO | A/G | rs947073006 | 5.8e-8 | -0.56 | Spleen |
| ABO | A/G | rs947073006 | 6.6e-8 | -0.29 | Nerve - Tibial |
| ABO | A/G | rs947073006 | 1.5e-7 | -0.29 | Artery – Coronary |
| SURF1 | A/G | rs947073006 | 1.5e-7 | 0.20 | Lung |
| SURF1 | A/G | rs947073006 | 1.8e-7 | 0.46 | Brain - Cerebellum |
| SURF1 | A/G | rs947073006 | 2.4e-7 | 0.24 | Skin - Not Sun Exposed (Suprapubic) |
| SURF1 | A/G | rs947073006 | 3.6e-7 | 0.21 | Nerve - Tibial |
| SURF1 | A/G | rs947073006 | 3.8e-7 | 0.23 | Breast - Mammary Tissue |
| SURF1 | A/G | rs947073006 | 5.6e-7 | 0.18 | Artery - Tibial |
| SURF1 | A/G | rs947073006 | 6.0e-7 | 0.22 | Skin - Sun Exposed (Lower leg) |
| SURF1 | A/G | rs947073006 | 8.4e-7 | 0.26 | Esophagus - Mucosa |
| SURF1 | A/G | rs947073006 | 1.3e-6 | 0.19 | Thyroid |
| ABO | A/G | rs947073006 | 1.7e-6 | -0.30 | Esophagus - Gastroesophageal Junction |
| ABO | A/G | rs947073006 | 5.1e-6 | -0.19 | Lung |
| SURF1 | A/G | rs947073006 | 1.2e-5 | 0.45 | Brain - Putamen (basal ganglia) |
| SURF1 | A/G | rs947073006 | 1.5e-5 | 0.088 | Whole Blood |
| ABO | A/G | rs947073006 | 2.1e-5 | -0.14 | Colon - Transverse |
| REXO4 | A/G | rs947073006 | 3.6e-5 | 0.15 | Cells - Cultured fibroblasts |
| ABO | A/G | rs947073006 | 4.7e-5 | -0.22 | Testis |
| MED22 | A/G | rs947073006 | 5.5e-5 | -0.088 | Whole Blood |
| SURF1 | A/G | rs947073006 | 8.6e-5 | 0.22 | Cells - Cultured fibroblasts |
| FGFR1 | C/T | rs881301 | 1.9e-41 | 0.43 | Whole Blood |
| RP11- 350N15.4 | C/T | rs881301 | 1.6e-11 | 0.27 | Whole Blood |
| FGFR1 | C/T | rs881301 | 3.0e-8 | 0.14 | Lung |
| RP11- 350N15.4 | C/T | rs881301 | 1.8e-6 | 0.16 | Lung |
| FGFR1 | C/T | rs881301 | 3.0e-6 | 0.13 | Cells - Cultured fibroblasts |
| FGFR1 | C/T | rs881301 | 4.8e-6 | 0.20 | Spleen |
| RP11-350N15.4 | C/T | rs881301 | 2.3e-5 | 0.16 | Muscle - Skeletal |
| FGFR1 | C/T | rs881301 | 3.2e-5 | 0.11 | Muscle - Skeletal |
| FGFR1 | C/T | rs881301 | 5.0e-5 | 0.15 | Testis |
| RP11-350N15.4 | C/T | rs881301 | 1.8e-4 | 0.13 | Esophagus - Mucosa |
| CHST9 | T/G | rs12607664 | 5.4e-5 | 0.35 | Brain - Cerebellum |

Locus, Gene symbol of the gene expression probe; EA,= effect allele; OA, other allele; SNP, rsID of the α-Klotho-associated SNPs; β, eQTL effect size.

SNP rs8176672 was queried using its synonym rs891364023; SNP rs532436 was queried using its synonym rs947073006; SNP rs7329055 (*KL*) was not part of any eQTL in GTEx v8. According to eQTLGen, rs7329055 is part of a significant *cis*-eQTL with *KL* (allele G: Z=4.8945, *p*=9.8606e-7).

## Supplementary Table S11: Colocalisation analyses between α-Klotho and eQTLs of the genes closest to the top GWAS SNPs

| SNP | Chr. | Position | Closest gene | Ensemble ID | N_SNPs_ | PP_H0_ | PP_H1_ | PP_H2_ | PP_H3_ | PP_H4_ |
| --- | --- | --- | --- | --- | --- | --- | --- | --- | --- | --- |
| rs881301 | 8 | 38332318 | FGFR1 | ENSG00000077782 | 1450 | 0.0% | 0.0% | 0.3% | 99.2% | 0.6% |
| rs8176672 | 9 | 136142185 | ABO | ENSG00000175164 | 2627 | 0.0% | 0.0% | 0.0% | 100.0% | 0.0% |
| rs532436 | 9 | 136149830 | ABO | ENSG00000175164 | 2627 | 0.0% | 0.0% | 0.0% | 100.0% | 0.0% |
| rs1056008 | 12 | 662838 | B4GALNT3 | ENSG00000139044 | 2745 | 0.0% | 0.0% | 0.0% | 0.2% | 99.8% |
| rs7333961 | 13 | 33533269 | KL | ENSG00000133116 | 1958 | 0.0% | 0.0% | 0.0% | 100.0% | 0.0% |

Note: SNP, Chr., and Position are the rsID, chromosome, and position (hg19) of the α-Klotho-associated SNPs.

Closest gene and Ensemble ID are the gene name and ID of the closest gene relative to the SNPs. N_SNPs_, PP_H0_, PP_H1_, PP_H2_, PP_H3_, and PP_H4_ are the number of SNPs used in the colocalization analysis and the posterior probabilities of the five possible hypotheses of the colocalization analysis: no effect for both (H0); with effect in one of the traits (H1 or H2); with effect in both traits but the two signals did not colocalize (H3); with effect in both traits and the two signals colocalize (H4).

No suitable data was available for SNP rs12607664 (locus on chr. 18) from the eQTLGen consortium.

# Supplementary Figures

## Supplementary Figure S1: Forest Plots


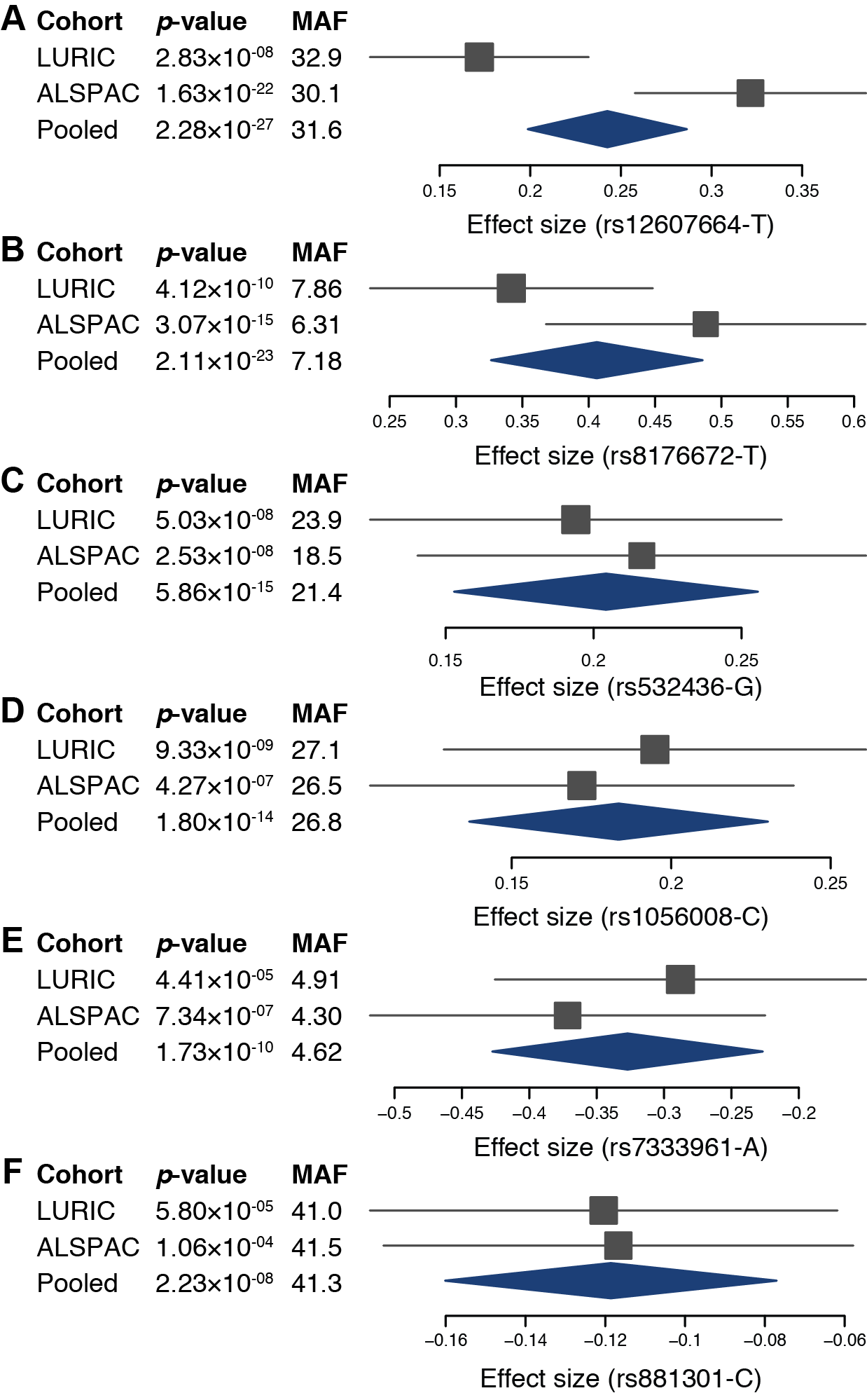


The table shows genetic associations with plasma α-Klotho levels for the six top=associated SNPs in the LURIC and ALSPAC cohorts separately and combined. MAF, minor allele frequency.

## Supplementary Figure S2: Open-chromatin results


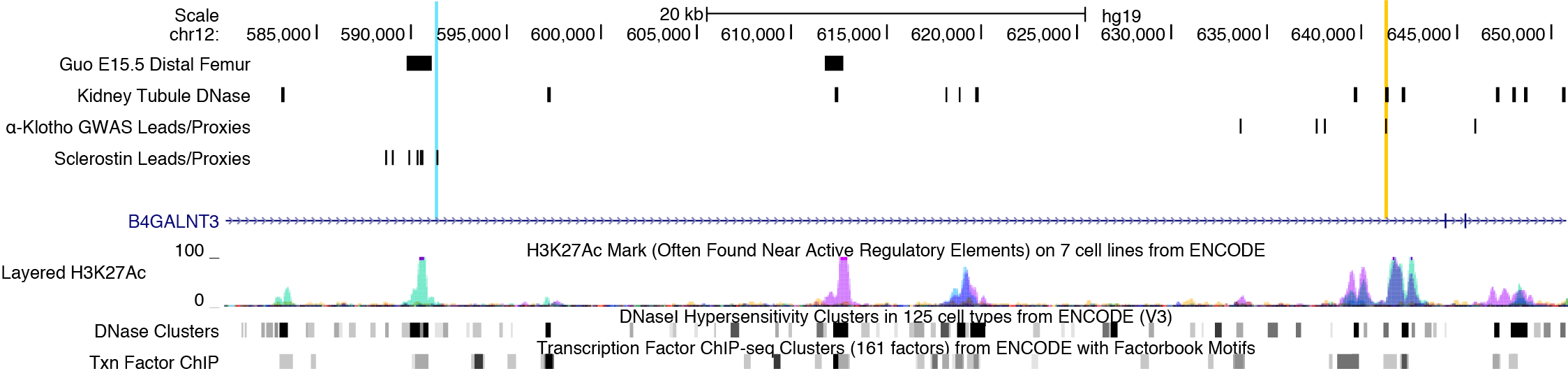


UCSC Genome Browser view of the *B3GALNT3* locus indicating the presence and regulatory activity of two distinct GWAS signals.

**Blue line to the left:** an ATAC-seq open-chromatin region from mouse E15.5 distal femur intersects proxy variants for rs215226, a lead variant identified in a sclerostin GWAS (39).

**Orange line to the right:** a DNase-I open-chromatin region from primary kidney tubule cell samples intersects the lead α-Klotho variant rs1009724.

Tracks below show additional ENCODE epigenetic data indicating regulatory activity in these two regions.

## Supplementary Figure S3: α-Klotho levels in *B4GALNT3* null mice


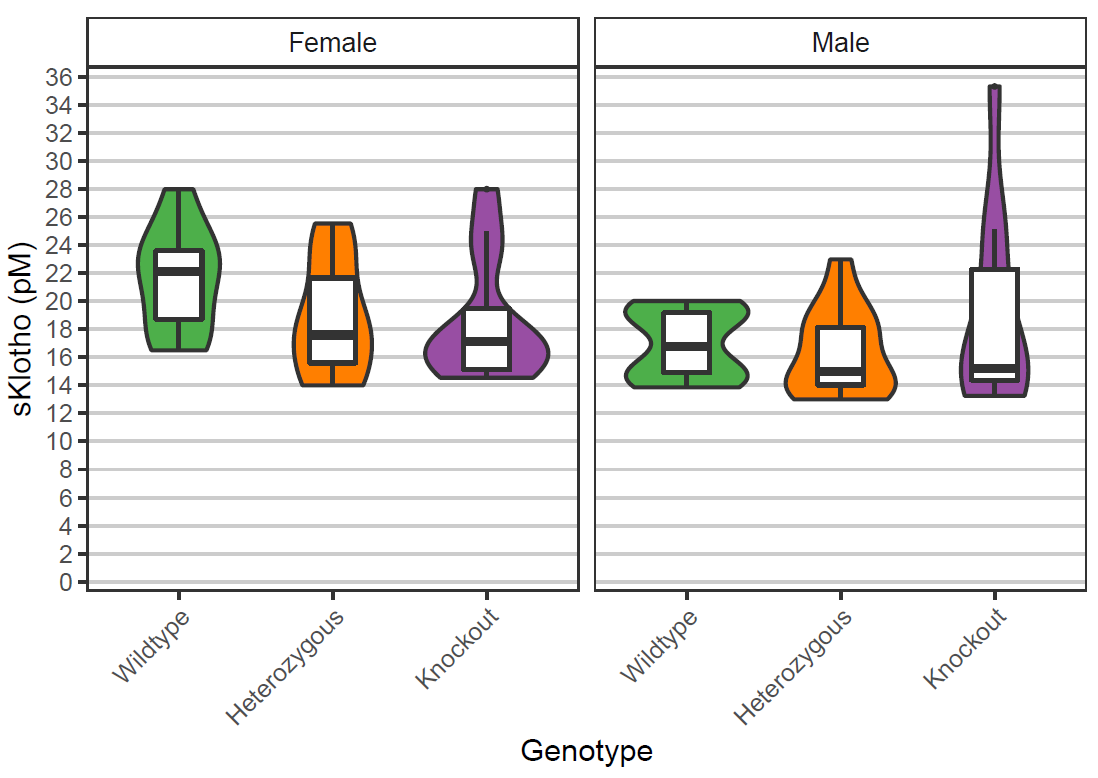


Box and whiskers and violin plots of soluble klotho levels (pM) in serum from *B4GALNT3* wildtype, heterozygous and knockout mice. The plot was generated in *R* using *ggplot2* and shows the median, inter-quartile range, overall range, and outliers.

Female wildtype n = 11; female heterozygous n = 11; female knockout; n = 12; male wildtype: n =14; male heterozygous: n = 13; male knockout: n = 11.
